# Single-crystal structure analysis of non-deuterated triglycine sulfate by neutron diffraction at 20 and 298 K: a new disorder model for the 298 K structure

**DOI:** 10.1107/S2056989022000858

**Published:** 2022-02-08

**Authors:** Yukana Terasawa, Takashi Ohhara, Sota Sato, Satoshi Yoshida, Toru Asahi

**Affiliations:** a School of Advanced Science and Engineering, Waseda University, 2-2 Wakamatsu-cho, Shinjuku-ku, Tokyo, 162-8480, Japan; b J-PARC Center, Japan Atomic Energy Agency, Shirakata 2-4, Tokai, Ibaraki, 319-1195, Japan; c Integrated Molecular Structure Analysis Laboratory, Department of Applied Chemistry, School of Engineering, The University of Tokyo, 7-3-1 Hongo, Bunkyo-ku, Tokyo, 113-8656, Japan; d Department of Applied Chemistry, School of Engineering, The University of Tokyo, 7-3-1 Hongo, Bunkyo-ku, Tokyo, 113-8656, Japan; e Faculty of Science and Engineering, Waseda University, 2-2 Wakamatsu-cho, Shinjuku-ku, Tokyo, 162-8480, Japan; f Research Organization for Nano & Life Innovation, Waseda University, 513 Wasedatsurumaki-cho, Shinjuku-ku, Tokyo, 162-0041, Japan

**Keywords:** triglycine sulfate, neutron diffraction, hydrogen atom, crystal structure

## Abstract

Precise single-crystal structure analyses of the title compound, bis­(glycinium) sulfate–glycine (1/1), 2C_2_H_6_NO_2_^+^·SO_4_^2−^·C_2_H_5_NO_2_ (or C_6_H_17_N_3_O_10_S), non-deuterated triglycine sulfate (HTGS) at 20 K and 298 K were undertaken using time-of-flight neutron diffraction data. At 20 K for the O—H⋯O hydrogen bond between the glycinium cation and the zwitterionic, unprotonated glycine mol­ecule that is associated with the ferroelectric behaviour of HTGS, O—H = 1.070 (3), H⋯O = 1.408 (3) [δ = 0.338 (4)], O⋯O = 2.4777 (15) Å and O—H⋯O = 179.0 (4)°, which is in good agreement with previous studies. Two reasonable structures for the same three atoms were refined for the 298 K dataset. One is a single-minimum potential-energy model, with O—H = 1.090 (12), H⋯O = 1.361 (12) [δ = 0.271 (17)], O⋯O = 2.450 (7) Å and O—H⋯O = 179.2 (10)°, having the H atom with a large ellipticity along the bond path between the O atoms. The other is a double-minimum potential-energy model having two H atom sites with occupancies of 0.876 (8) and 0.124 (8): for the major occupancy component, O—H = 1.065 (12), H⋯O = 1.387 (12), O⋯O = 2.451 (7) Å and O—H⋯O = 178.2 (11)° and for the minor component, O—H = 1.06 (4), H⋯O = 1.41 (4), O⋯O = 2.451 (7) Å and O—H⋯O = 166 (2)°. These models did not show any significant differences in *R* factors. In addition, the unit-cell parameters and other structural parameters of HTGS did not show any major differences compared to those of partially deuterated TGS and fully deuterated TGS for both 20 K and 298 K.

## Abstract 2

A precise crystal-structure analysis using a neutron diffractometer with high-power neutron sources at the J-PARC facility has been performed on non-deuterated triglycine sulfate at 20 K and 298 K and a new double-potential-well disorder model for the O—H⋯O hydrogen bond in the 298 K structure is proposed.

## Chemical context

Triglycine sulfate, 2(C_2_H_6_NO_2_)^+^·(C_2_H_5_NO_2_)·(SO_4_)^2–^ (TGS), is a hydrogen-bond ferroelectric material (Matthias *et al.* 1956 ▸) exhibiting a second-order and order–disorder-type ferroelectric phase transition at a Curie temperature (*T*_C_) of 322 K (Triebwasser, 1958 ▸). The TGS structure belongs to the point group *C*_2*h*_ and the space group *P*2_1_/*m* in the paraelectric phase and *C*_2_ and *P*2_1_ in the ferroelectric phase, respectively (Wood & Holden, 1957 ▸). Because of its high pyroelectricity, TGS has long been used as a material for pyroelectric sensors. Therefore, determining the crystal structure of TGS is essential for understanding such physical properties.

The atomic coordinates, except for those of the hydrogen atoms, of TGS at room temperature were first determined using single-crystal X-ray diffraction (Hoshino *et al.*, 1959 ▸). The study assumed the presence of one neutral glycine mol­ecule (C_2_H_5_NO_2_) exhibiting a zwitterionic configuration, and two monoprotonated glycinium ions (C_2_H_6_NO_2_^+^), from the detailed analysis of the bond lengths and angles of the glycine mol­ecules. The authors also proposed a hydrogen-bonding scheme and pointed out that the hydrogen atom that lies between the oxygen atom of the carboxyl group in the glycine III cation (GIII) and the O atom in the glycine II mol­ecule (GII) plays a crucial role in the dipole reversal. Many structural studies on TGS have subsequently been conducted (see *Database survey*): most of them were X-ray diffraction studies, but some of them were neutron diffraction studies. The atomic coordinates of non-deuterated TGS (hereinafter, designated as HTGS in place of TGS), including those of the hydrogen atoms at room temperature, were first revealed using single-crystal neutron diffraction (Padmanabhan & Yadav, 1971 ▸) and the atomic arrangements including hydrogen atoms of the zwitterion and glycinium ions were directly observed. The neutron diffraction experiment revealed that the hydrogen atom forming the O—H⋯O hydrogen bond between the GIII and GII species was closer to the GIII O atom compared to that in GII. This result agreed with that obtained by Hoshino *et al.* (1959 ▸). The structure refinement of HTGS with an applied external electric field at 298 K revealed the placement of all the hydrogen atoms and the unambiguous definition of the hydrogen-bonding scheme in an ordered domain structure (Kay & Kleinberg, 1973 ▸).

Crystal-structure refinements of partially deuterated TGS (DTGS), where deuterium replaced the H atoms except for the hydrogen atoms of the methyl­ene (CH_2_) group in each glycine mol­ecule and those in sulfuric acid mol­ecules at 40 K and 180 K (Protas *et al.*, 1997 ▸) showed that the refined structures were consistent with those of the HTGS reported by Kay & Kleinberg (1973 ▸). Protas *et al.* (1997 ▸) also observed that HTGS and DTGS in the ferroelectric phase had a consistent structure from 40 K to 298 K. The deuterium atom lying between GIII and GII was ∼0.40 Å closer to the O atom of the carboxyl group of GIII than that of GII at both temperatures. In contrast, the crystal-structure refinement of HTGS at room temperature showed positional disorder over two adjacent sites of the amino group in glycinium cation I (GI) (Choudhury & Chitra, 2008 ▸). However, this is not in agreement with the refined structure of HTGS reported by Padmanabhan & Yadav (1971 ▸) where the GI species was analysed as an ordered structure.

In the crystal structure of fully deuterated TGS (FDTGS), all the hydrogen atoms in the glycine mol­ecules and sulfuric acid mol­ecules are substituted by deuterium atoms: the crystal structures did not show major changes between 20 K and 295 K (Hudspeth *et al.*, 2013 ▸). The unit-cell parameters of these FDTGS structures were consistent with those of HTGS (Kay & Kleinberg, 1973 ▸; Choudhury & Chitra, 2008 ▸) and DTGS (Protas *et al.*, 1997 ▸).

Structural analysis of DTGS at 40 K and FDTGS at 20 K have been undertaken by Protas *et al.* (1997 ▸) and Hudspeth *et al.* (2013 ▸), respectively, as mentioned above. However, a precise structural analysis of HTGS including hydrogen atoms at low temperatures has not been reported. Furthermore, two different structures of HTGS at ∼298 K were reported: one is an ordered structure by Padmanabhan & Yadav (1971 ▸) and the other is a disordered structure by Choudhury & Chitra (2008 ▸). 
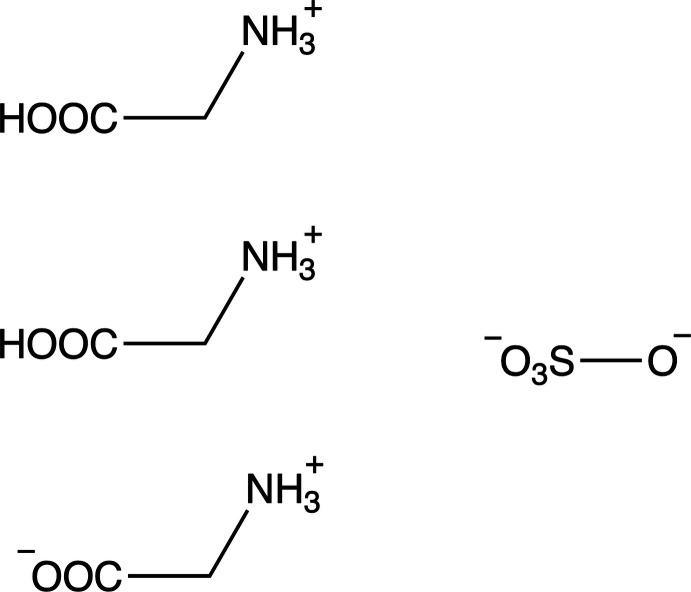


With this motivation, in this study, single-crystal neutron diffraction of HTGS has been conducted at 20 K and 298 K in the ferroelectric phase. The single crystal neutron diffractometer SENJU (Ohhara *et al.* 2016 ▸) at the J-PARC facility, which enables us to measure multiple Bragg reflections with high efficiency at low temperatures by combining high-power neutron sources and a time-of-flight Laue diffraction method, has firstly determined the precise crystal structure of HTGS at 20 K under suppression of thermal vibrations, including the atomic coordinates of the hydrogen atoms. Furthermore, a new structural model of HTGS at 298 K is proposed in addition to the structural model reported previously.

## Structural commentary

### Structural Refinement of HTGS at 20 K

**Figure 1:**
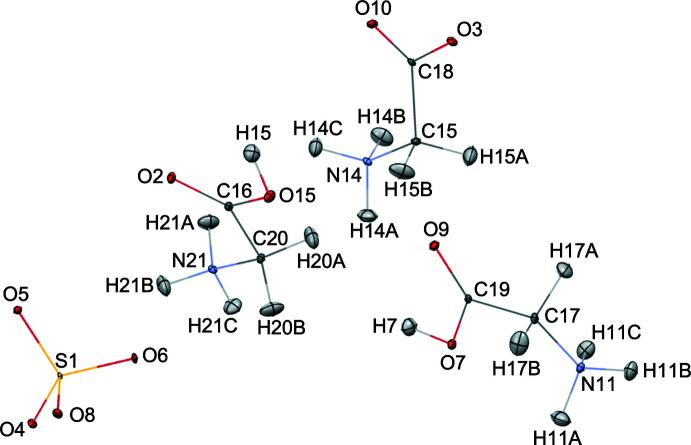
The mol­ecular structure of HTGS at 20 K showing 50% displacement ellipsoids for all atoms.

The refined structures at 20 K are shown in Figs. 1 ▸ and 2 ▸. It was confirmed that the GII C_2_H_5_NO_2_ glycine mol­ecule containing C15 exhibits the neutral zwitterion configuration, and the other two GI and GIII glycine moieties (C17 and C20, respectively) exist as monoprotonated C_2_H_6_NO_2_^+^ glycinium ions. The most significant feature of these glycine/glycinium species are the N—C—C—O(H) torsion angles (Terasawa *et al.* 2021 ▸), *viz*.: 21.1 (1)° for N11—C17—C19—O7, −1.5 (1)° for N14—C15—C18—O10 and −1.4 (1)° for N21—C20—C16—O2. The sulfate ion shows its expected tetra­hedral shape with bond distances of 1.480 (2) Å (S1—O4), 1.470 (2) Å (S1—O5), 1.477 (2) Å (S1—O6) and 1.472 (2) Å (S1—O8) and bond angles of 110.3 (1)° (O4—S1—O5), 107.9 (1)° (O4—S1—O6), 108.7 (1)° (O4—S1—O8), 109.7 (1)° (O5—S1—O6), 110.6 (1)° (O5—S1—O8) and 109.7 (1)° (O6—S1—O8). The slight differences among these distances and angles may arise because of the different hydrogen bonds accepted by these O atoms. Numerous N—H⋯O and O—H⋯O hydrogen bonds (see supporting information) are formed between the glycine or glycinium species and the sulfate ions; four N—H⋯O hydrogen bonds and one O—H⋯O hydrogen bond are formed by GI, five N—H⋯O hydrogen bonds are formed by GII and five N—H⋯O hydrogen bonds with the sulfate ion and one O—H⋯O hydrogen bond to the glycine mol­ecule is formed by GIII.

The lattice constants and the key O15—H15⋯O3^i^ [symmetry code: (i) 3 − *x*, − 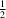 + *y*, 2 − *z* for the present study] bond lengths for HTGS, DTGS and FDTGS at low temperature are listed in Table 1 ▸. The parameters do not show any major differences, and H15 is 0.338 (4) Å closer to atom O15 in GIII than O3 in GII. This result shows good agreement with the data previously reported for DTGS (Protas *et al.* 1997 ▸), thus it may be concluded that the inter­molecular distances and angles do not change significantly upon deuteration.

### Structural Refinement of HTGS at 298 K

**Figure 3:**
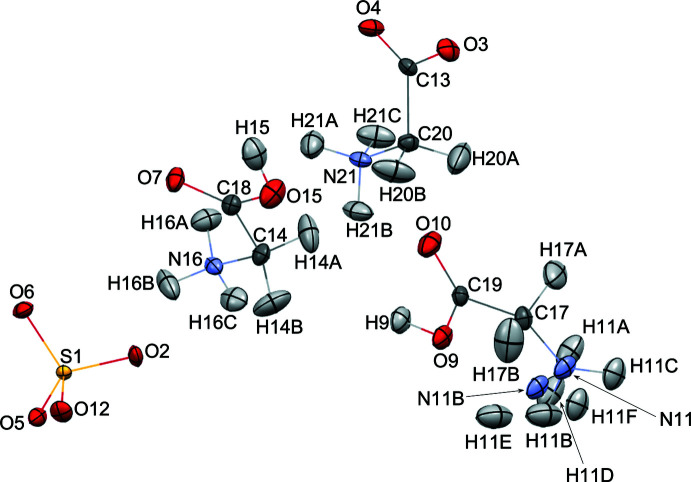
The mol­ecular structure of HTGS at 298 K (model 1) showing 50% displacement ellipsoids for all atoms.

**Figure 5:**
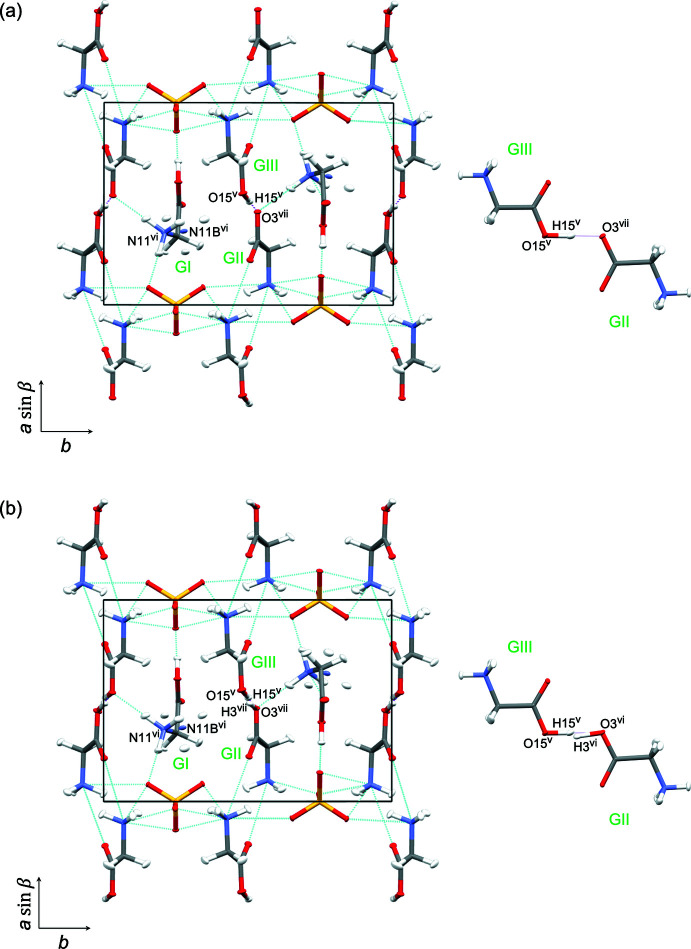
[001] projection of the unit cell and detail of the hydrogen bond between GII and GIII of HTGS at 298 K with hydrogen bonds shown as dashed lines. The short O15—H15⋯O3 bonds are shown as pink dashed lines. Atoms N11 and N11*B* are disordered with occupancies of 87.5%/12.5%. Model 1 (*a*); a single-minimum potential energy model for H5; model 2 (*b*); a double-minimum potential-energy model for H15 and H3. Symmetry codes: (v) 1 − *x*, − 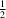 + *y*, 1 − *z*; (vi) 1 − *x*, − 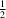 + *y*, 2 − *z*; (vii) *x*, −1 + *y*, 1 + *z*.

The refined structures at 298 K are shown in Figs. 3 ▸, 4 ▸ and 5 ▸. The crystallographic symmetry, the contents of the asymmetric unit, and the features of the mol­ecular structures are consistent with those for the 20 K structure apart the disordered N11/N11*B* amino group [refined site occupancies = 0.874 (8):0.126 (8)] in the GI cation and the O—H⋯O association for GIII and GII. Two models were refined considering the H atom between O15 in GIII and O3 in GII. For one model (298 K model 1), the H15 atom was refined with a large ellipticity along the bond path between O15 and O3 as a single minimum potential energy structure [Fig. 5 ▸(*a*)]. A double-minimum potential-energy structure could be deduced because the distance between O15 and O3^i^ [symmetry code: (i) 1 − *x*, − 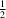 + *y*, −*z* for the present study] did not increase with an increase in the temperature; thus for the other model (298 K model 2), a pair of hydrogen atoms were refined along the bond path between O15 and O3^i^, the double-minimum potential structure [Fig. 5 ▸(*b*)].

The key parameters for the O15—H15⋯O3^i^ hydrogen bond at 298 K are summarized in Table 2 ▸. The residuals for models 1 and 2 (Table 3 ▸) are almost identical: model 2 has one more variable parameter than model 1 (358 compared to 357). For model 1, H15 is 0.271 (17) Å closer to O15 in GIII than O3^i^ in GII. On the other hand, the distance between O15 and H15 [1.090 (12) Å] is almost the same as that at 20 K despite there being no distance restraint for the H15⋯O3^i^ separation. Therefore, the mixed structure (model 2) of the major ferroelectric phase and minor paraelectric phase is strongly suggested, because the occupancies of N11 and N11*B* and H15 and H3^i^ are related by symmetry.

The unit-cell parameters and bond lengths for HTGS, DTGS, and FDTGS at 298 K are listed in Table 2 ▸. The lattice parameters did not show any major differences and this result shows good agreement with that previously reported for DTGS (Protas *et al.*, 1997 ▸). We may conclude that the inter­molecular distances and angles do not change significantly upon deuteration.

In the previous studies using single-crystal neutron diffraction, Kay & Kleinberg (1973 ▸) proposed an ordered structure of HTGS because the domains were oriented by applying an external electric field. Hudspeth & Goossens (2012 ▸) proposed an ordered structure for FDTGS because *T*_C_ for FDTGS increased by approximately 12 K compared to HTGS. Choudhury & Chitra (2008 ▸) proposed a disordered structure for the GI amino group with unequal occupancies of N11 (88%) and N11*B* (12%); this occupancy ratio is in excellent agreement with the results in this study. For the hydrogen atom between the oxygen atom of the carboxyl group in GIII and that in the GII, the O⋯O distance was 2.470 (9) Å, and the H atom was approximately 0.241 Å closer to the GIII O atom than that in GII. They concluded that the structure of HTGS at room temperature has a single minimum potential energy in the O—H⋯O hydrogen-bond path between GIII and GII. In this study, two reasonable structures were refined as a single-minimum potential-energy model and a double-minimum model without any significant differences. Therefore, we conclude that there is a significant possibility of a double-minimum potential-energy model for HTGS at 298 K.

## Supra­molecular features

Hydrogen bonds in the refined structures were consistent with those reported previously (see supporting information) and no additional inter­molecular inter­actions were found. Therefore, the 20 K and 298 K structures form essentially the structural motif of a three-dimensional network of N—H⋯O and O—H⋯O hydrogen bonds between glycinium cations, glycine mol­ecules and sulfate ions.

## Database survey

The Cambridge Structural Database (Version 5.42, update of November 2020; Groom *et al.* 2016 ▸) was searched for structures of triglycine sulfate and it returned no fewer than 29 hits: six of these records are structures obtained using neutron diffraction. The lattice constants of these structures are consistent with those of this study. The ionic states of glycine and the sulfate ion for five structures obtained using single-crystal neutron data are consistent with those for this study in which one neutral, zwitterionic glycine mol­ecule and two monoprotonated glycinium ions occur [CSD refcodes TGLYSU01 (Protas *et al.*, 1997 ▸); TGLYSU02 (Padmanabhan & Yadav, 1971 ▸); TGLYSU03 (Protas *et al.*, 1997 ▸); TGLYSU11 (Kay & Kleinberg, 1973 ▸); and TGLYS25 (Cheng *et al.*, 1986 ▸)]. In contrast, hydrogen atoms were not assigned in some of the structures obtained using X-ray diffraction: refcodes TGLYSU (Hoshino *et al.*, 1959 ▸); TGLYSU13 (Itoh & Mitsui, 1973 ▸); TGLYSU28 (Choudhury & Chitra, 2008 ▸); TGLYSU29 (Kawasaki *et al.*, 2021 ▸) and TGLYSU30 (Kawasaki *et al.*, 2021 ▸). Furthermore, in several structures, some hydrogen atoms are missing: refcodes TGLYSU04 (Fletcher *et al.*, 1976 ▸); TGLYSU07 (Solans *et al.*, 1985); TGLYSU15 (Itoh & Mitsui, 1973 ▸); TGLYSU21, TGLYSU22, TGLYSU23 (Kolontsova *et al.*, 1990 ▸). In one structure, HSO_4_^−^ ions were proposed to be present: refcode TGLYSU04 (Fletcher *et al.*, 1976 ▸).

## Synthesis and crystallization

The HTGS crystals were grown in an aqueous solution by the slow evaporation method at ∼293 K. Glycine (13.06 g; FUJIFILM Wako Pure Chemical Corporation; purity ≥ 99.0%) and sulfuric acid (3.1 ml; FUJIFILM; molar ratio 3:1) was added to 50 ml of water in a 100 ml beaker. They were dissolved by heating at ∼313 K with a 300 r.p.m. magnetic stirrer. After completely dissolving them, plastic films were double-wrapped around the beaker, and some holes were knocked in the films to evaporate the water slowly. The beaker was left to stand at ∼293 K. HTGS was crystallized after approximately a month, and then the solution was filtered. The collected crystals were dried in a desiccator at ∼293 K.

## Refinement

Crystal data, data collection, and structural refinement details are summarized in Table 3 ▸. All data were collected using the single-crystal neutron diffractometer SENJU (Ohhara *et al.*, 2016 ▸) at beamline BL18 of the Materials and Life Science Facility, Japan Proton Accelerator Research Complex. The crystal (colourless cube, ∼2.8 mm edge length) mounted on an aluminum pin was cooled to 20 K in a closed-cycle helium cryostat. The crystal was surrounded by 41 two-dimensional scintillation area detectors during the data collection. The same crystal was used for the measurement at 298 K after warming to room temperature. Three-dimensional data of (*x*, *y*, *λ*) were measured in 16 different orientations for each dataset. The measurement time was 1.5 h for one orientation; the raw data were processed using *STARGazer* (Ohhara *et al.* 2009 ▸) to generate HKLF files and visualize (*x*, *y*) slice maps and merged TOF profiles.

**Figure 6:**
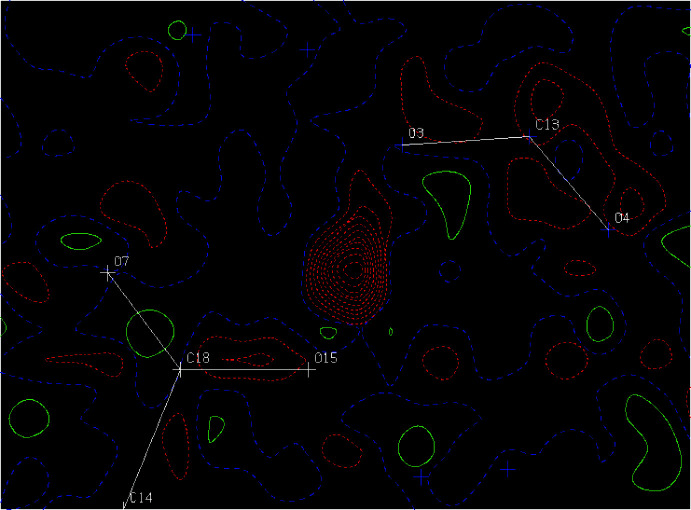
A difference scattering density map for the 298 K structure without the H atom between O15 and O3. The red dotted lines and green solid lines show negative and positive density distribution, respectively. A nuclear density distribution with a large ellipticity along the bond path between O15 and O3 is observed.

*SHELXL2018* (Sheldrick 2015*b* ▸) was used for least-squares refinements with neutron scattering lengths (fm) of 2.847 (S), 5.805 (O), −3.741 (H), 9.360 (N) and 6.648 (C). A reported structure determined by single-crystal X-ray diffraction (Hoshino *et al.*, 1959 ▸) was used as the initial structural model. All atoms, including hydrogen atoms, were refined with *U*_ij_ values. For the 298 K data, the refinement was initially performed without the hydrogen atom(s) between O15 and O3 to minimize the model dependence. A nuclear density distribution (Fig. 6 ▸) with a large ellipticity along the bond path between O15 and O3 was observed. One hydrogen atom was assigned to this position and refined as a single-minimum potential-energy model (298 K model 1). In 298 K model 2, two hydrogen atoms (H15 and H3) with the restrictions listed below were included: (i) H15 and H3 were refined anisotropically and constrained to have the same displacement factors; (ii) O15 and H15 and O3 and H3 were restrained to have the same distances; (iii) the occupancies of H15 for H3 were linked to those of N11 and N11*B*.

## Supplementary Material

Crystal structure: contains datablock(s) global, 20K, 298KModel1, 298KModel2. DOI: 10.1107/S2056989022000858/hb8004sup1.cif

Structure factors: contains datablock(s) 20K. DOI: 10.1107/S2056989022000858/hb800420Ksup2.hkl

Supporting information file. DOI: 10.1107/S2056989022000858/hb800420Ksup5.cml

Structure factors: contains datablock(s) 298KModel1. DOI: 10.1107/S2056989022000858/hb8004298KModel1sup3.hkl

Supporting information file. DOI: 10.1107/S2056989022000858/hb8004298KModel1sup6.cml

Structure factors: contains datablock(s) 298KModel2. DOI: 10.1107/S2056989022000858/hb8004298KModel2sup4.hkl

Supporting information file. DOI: 10.1107/S2056989022000858/hb8004298KModel2sup7.cml

CCDC references: 2144164, 2144163, 2144162

Additional supporting information:  crystallographic information; 3D view; checkCIF report

## Figures and Tables

**Figure 2:**
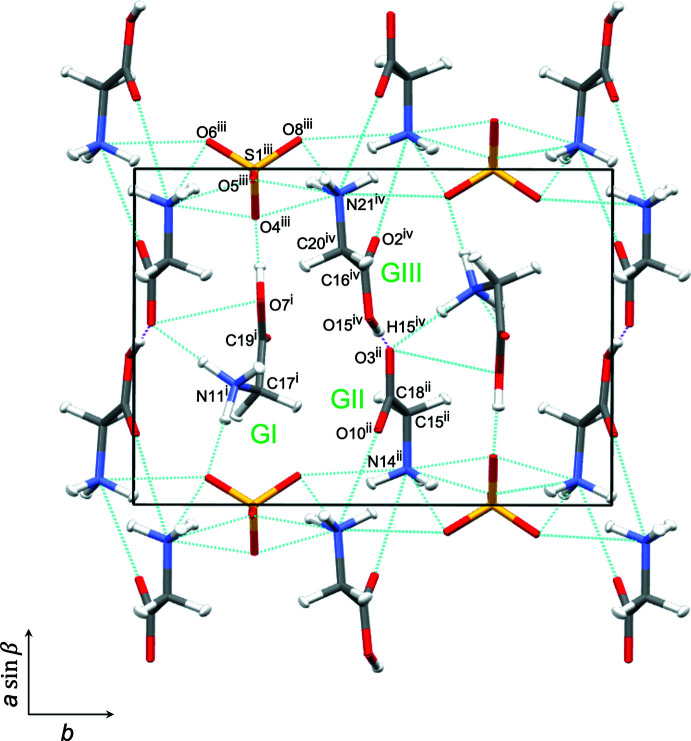
[001] projection of the unit cell of HTGS at 20 K with hydrogen bonds shown as dashed lines. The O15—H15⋯O3 hydrogen bonds are shown as pink dashed lines. Glycine mol­ecules are represented as glycine I (GI), glycine II (GII), and glycine III (GIII), according to Hoshino *et al.* (1959 ▸). Symmetry codes: (i) −1 + *x*, *y*, 1 + *z*; (ii) −1 + *x*, *y*, *z*; (iii) 1 − *x*, 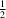 + *y*, 1 − *z*, (iv) 2 − *x*, − 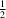 + *y*, 2 − *z*.

**Figure 4:**
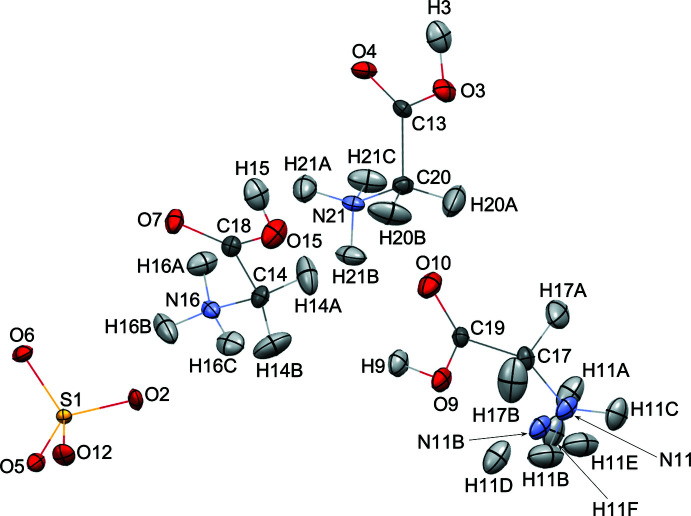
The mol­ecular structure of HTGS at 298 K (model 2) showing 50% displacement ellipsoids for all atoms.

**Table 1:** Lattice constants, inter­atomic distances and angles for HTGS, DTGS, and FDTGS at low temperatures

	This study	Protas *et al.* (1997 ▸)	Hudspeth *et al.* (2013 ▸)
	HTGS	DTGS	FDTGS
Temperature (K)	20 (2)	40	20 (2)
*a* (Å)	9.3946 (8)	9.406 (5)	9.409 (2)
*b* (Å)	12.5338 (11)	12.614 (5)	12.558 (3)
*c* (Å)	5.6630 (4)	5.654 (5)	5.673 (1)
*β* (Å)	110.500 (7)	110.49 (2)	110.44 (2)
*V* (Å^3^)	624.59 (9)	628.4 (7)	628.2 (2)
O15⋯O3^i^ (Å)	2.4777 (15)	2.486 (5)	–
O15—H(D)15 (Å)	1.070 (3)	1.041 (5)	–
H(D)15⋯O3^i^ (Å)	1.408 (3)	1.445 (6)	–
O15—H(D)15⋯O3^i^ (°)	179.0 (4)	178.4 (6)	–

Symmetry code for HTGS in this study: (i) 3 − *x*, − 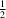 + *y*, 2 − *z*.

**Table 2:** Lattice constants, inter­atomic distances and angles at disordered atoms for HTGS and FDTGS at ∼298 K

	This study (model 1)	This study (model 2)	Kay *et al.* (1973 ▸)	Choudhury & Chitra (2008 ▸)	Hudspeth *et al.* (2013 ▸)
	HTGS	HTGS	HTGS	HTGS	FDTGS
Temperature (K)	298 (2)	298 (2)	298	RT	295
*a* (Å)	9.3910 (14)	9.3910 (14)	9.417	9.416 (7)	9.413 (2)
*b* (Å)	12.6021 (18)	12.6021 (18)	12.643	12.643 (1)	12.629 (2)
*c* (Å)	5.7125 (7)	5.7125 (7)	5.735	5.734 (3)	5.716 (1)
*β* (Å)	110.306 (13)	110.306 (13)	110.4	110.33 (3)	110.30 (2)
*V* (Å^3^)	634.04 (16)	634.04 (16)	639.98	640.09	637.3 (2)
O15⋯O3^i^ (Å)	2.450 (7)	2.451 (7)	2.50	2.470 (9)	–
O15—H(D)15 (Å)	1.090 (12)	1.065 (12)	1.10	1.115 (12)	1.077 (6)
H(D)15⋯O3^i^ (Å)	1.361 (12)	1.387 (12)	1.36	1.356 (11)	–
H(D)3—O3 (Å)	–	1.06 (4)	–	–	–
O15—H(D)15⋯O3^i^ (°)	179.2 (10)	178.2 (11)	176 (2)	177.3 (9)	–

Symmetry code for HGTS in this study: (i) 1 − *x*, − 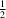 + *y*, −*z*.

**Table 3:** Experimental details

	20 K	298 K model 1	298 K model 2
Crystal data			
Chemical formula	2C_2_H_6_NO_2_^+^·SO_4_^2−^·C_2_H_5_NO_2_	2C_2_H_6_NO_2_^+^·SO_4_^2−^·C_2_H_5_NO_2_	2C_2_H_6_NO_2_^+^·SO_4_^2−^·C_2_H_5_NO_2_
*M*_r_	323.28	323.28	323.28
Crystal system, space group	Monoclinic, *P*2_1_	Monoclinic, *P*2_1_	Monoclinic, *P*2_1_
Temperature (K)	20	298	298
*a*, *b*, *c* (Å)	9.3946 (8), 12.5338 (11), 5.6630 (4)	9.3910 (14), 12.6021 (18), 5.7125 (7)	9.3910 (14), 12.6021 (18), 5.7125 (7)
β (°)	110.500 (7)	110.306 (13)	110.306 (13)
*V* (Å^3^)	624.59 (9)	634.04 (16)	634.04 (16)
*Z*	2	2	2
Radiation type	Neutrons, λ = 1 Å	Neutrons, λ = 1 Å	Neutrons, λ = 1 Å
μ (mm^−1^)	0.49	0.49	0.49
Crystal size (mm)	2.80 × 2.80 × 2.80	2.80 × 2.80 × 2.80	2.80 × 2.80 × 2.80

Data collection			
Diffractometer	Time-of-flight Laue-type single crystal neutron diffractometer	Time-of-flight Laue-type single crystal neutron diffractometer	Time-of-flight Laue-type single crystal neutron diffractometer
No. of measured, independent and observed [*I* > 2σ(*I*)] reflections	40510, 10169, 33150	14190, 3132, 10685	14190, 3132, 10685
(sin θ/λ)_max_ (Å^−1^)	0.998	0.994	0.994

Refinement			
*R*[*F* ^2^ > 2σ(*F* ^2^)], *wR*(*F* ^2^), *S*	0.073, 0.193, 1.04	0.080, 0.209, 1.05	0.080, 0.209, 1.05
No. of reflections	40510	14190	14190
No. of parameters	350	357	358
No. of restraints	1	1	8
H-atom treatment	All H-atom parameters refined	All H-atom parameters refined	All H-atom parameters refined
Δρ_max_, Δρ_min_ (e Å^−3^)	3.87, −6.29	3.50, −6.06	1.50, −1.70
Absolute structure	Indeterminate for a neutron structure	Indeterminate for a neutron structure	Indeterminate for a neutron structure

Computer programs: *STARGazer* (Ohhara *et al.*, 2009 ▸), *SHELXL2018/3* (Sheldrick, 2015 ▸), *Mercury* (Macrae *et al.*, 2020 ▸), *PLATON* (Spek, 2020 ▸) and *publCIF* (Westrip, 2010 ▸).

## supplementary crystallographic information

### Bis(glycinium) sulfate–glycine (1/1) (20K) Crystal data

**Table:**

2C_2_H_6_NO_2_^+^·SO_4_^2^^−^·C_2_H_5_NO_2_	*F*(000) = 130.536
*M**_r_* = 323.28	*D*_x_ = 1.719 Mg m^−^^3^
Monoclinic, *P*2_1_	Neutrons radiation, λ = 1 Å
Hall symbol: P2yb	Cell parameters from 8174 reflections
*a* = 9.3946 (8) Å	θ = 6.6–83.3°
*b* = 12.5338 (11) Å	µ = 0.49 mm^−^^1^
*c* = 5.6630 (4) Å	*T* = 20 K
β = 110.500 (7)°	Block, colorless
*V* = 624.59 (9) Å^3^	2.80 × 2.80 × 2.80 mm
*Z* = 2	

### Bis(glycinium) sulfate–glycine (1/1) (20K) Data collection

**Table:**

Time-of-flight Laue-type single crystal neutron diffractometer	33150 reflections with *I* > 2σ(*I*)
Radiation source: spallation neutron	*R*_int_ = N/A
Detector resolution: 4 pixels mm^-1^	θ_max_ = 86.1°, θ_min_ = 7.5°
time–of–flight Laue method scans	*h* = −23→23
40510 measured reflections	*k* = −31→31
10169 independent reflections	*l* = −13→14

### Bis(glycinium) sulfate–glycine (1/1) (20K) Refinement

**Table:**

Refinement on *F*^2^	All H-atom parameters refined
Least-squares matrix: full	*w* = 1/[σ^2^(*F*_o_^2^) + (0.1332*P*)^2^ + 0.072*P*] where *P* = (*F*_o_^2^ + 2*F*_c_^2^)/3
*R*[*F*^2^ > 2σ(*F*^2^)] = 0.073	(Δ/σ)_max_ = 0.001
*wR*(*F*^2^) = 0.193	Δρ_max_ = 3.87 e Å^−^^3^
*S* = 1.04	Δρ_min_ = −6.28 e Å^−^^3^
40510 reflections	Extinction correction: SHELXL2018/3 (Sheldrick 2015)
350 parameters	Extinction coefficient: 0.119 (3)
1 restraint	Absolute structure: Indeterminate for a neutron structure

### Bis(glycinium) sulfate–glycine (1/1) (20K) Special details

**Table:**

Geometry. All esds (except the esd in the dihedral angle between two l.s. planes) are estimated using the full covariance matrix. The cell esds are taken into account individually in the estimation of esds in distances, angles and torsion angles; correlations between esds in cell parameters are only used when they are defined by crystal symmetry. An approximate (isotropic) treatment of cell esds is used for estimating esds involving l.s. planes.
Refinement. reflns_Friedel_fraction is defined as the number of unique Friedel pairs measured divided by the number that would be possible theoretically, ignoring centric projections and systematic absences.'

### Bis(glycinium) sulfate–glycine (1/1) (20K) Fractional atomic coordinates and isotropic or equivalent isotropic displacement parameters (Å^2^)

**Table:**

	*x*	*y*	*z*	*U*_iso_*/*U*_eq_	
S1	0.9987 (2)	0.25000 (15)	0.2280 (4)	0.00149 (18)	
O3	1.46271 (12)	0.96537 (9)	0.8104 (2)	0.00493 (13)	
O2	1.20940 (13)	0.49653 (10)	0.7552 (2)	0.00564 (13)	
O4	0.85445 (12)	0.24777 (9)	0.0101 (2)	0.00407 (11)	
O5	0.96806 (13)	0.25228 (9)	0.4652 (2)	0.00438 (12)	
O6	1.08235 (12)	0.34725 (8)	0.2072 (2)	0.00397 (12)	
O7	1.39163 (12)	0.73620 (10)	−0.0953 (2)	0.00519 (13)	
H7	1.2917 (3)	0.7379 (3)	−0.0588 (6)	0.0160 (4)	
O8	1.08828 (12)	0.15498 (8)	0.2172 (2)	0.00403 (12)	
O9	1.50274 (14)	0.72358 (11)	0.3230 (2)	0.00628 (14)	
O10	1.22125 (13)	0.98973 (10)	0.7827 (2)	0.00528 (13)	
O15	1.44257 (13)	0.50951 (11)	0.7343 (2)	0.00643 (14)	
H15	1.4843 (4)	0.4899 (3)	0.9306 (6)	0.0162 (4)	
N11	1.64288 (8)	0.78825 (6)	−0.17333 (14)	0.00468 (8)	
H11A	1.5930 (5)	0.7383 (3)	−0.3227 (7)	0.0206 (5)	
H11B	1.7487 (3)	0.8093 (3)	−0.1799 (7)	0.0167 (4)	
H11C	1.5753 (4)	0.8567 (3)	−0.2021 (6)	0.0165 (4)	
N14	1.10601 (8)	0.92901 (5)	0.29966 (13)	0.00407 (8)	
H14A	1.0741 (4)	0.9005 (3)	0.1183 (6)	0.0189 (5)	
H14B	1.0637 (4)	1.0044 (3)	0.2961 (8)	0.0202 (6)	
H14C	1.0593 (4)	0.8781 (3)	0.3977 (7)	0.0186 (5)	
N21	1.07375 (7)	0.57354 (5)	0.28504 (13)	0.00395 (8)	
H21A	1.0403 (4)	0.6295 (3)	0.3907 (7)	0.0184 (5)	
H21B	1.0241 (4)	0.5017 (3)	0.2905 (7)	0.0194 (5)	
H21C	1.0335 (4)	0.6013 (3)	0.1022 (6)	0.0165 (4)	
C15	1.27299 (11)	0.92965 (8)	0.42088 (19)	0.00464 (11)	
H15A	1.3210 (5)	0.9838 (4)	0.3185 (7)	0.0231 (7)	
H15B	1.3161 (5)	0.8502 (3)	0.4096 (8)	0.0227 (7)	
C16	1.29570 (10)	0.51955 (7)	0.64503 (18)	0.00353 (10)	
C17	1.65950 (10)	0.73969 (8)	0.07314 (19)	0.00453 (11)	
H17A	1.7376 (4)	0.7882 (3)	0.2221 (7)	0.0200 (6)	
H17B	1.7072 (5)	0.6594 (3)	0.0824 (9)	0.0217 (6)	
C18	1.31907 (10)	0.96459 (7)	0.69381 (17)	0.00311 (10)	
C19	1.50928 (10)	0.73247 (7)	0.11344 (18)	0.00345 (10)	
C20	1.24065 (11)	0.56505 (8)	0.38171 (19)	0.00430 (10)	
H20A	1.2898 (4)	0.6438 (3)	0.3846 (8)	0.0207 (6)	
H20B	1.2774 (4)	0.5157 (4)	0.2554 (7)	0.0215 (6)	

### Bis(glycinium) sulfate–glycine (1/1) (20K) Atomic displacement parameters (Å^2^)

**Table:**

	*U*^11^	*U*^22^	*U*^33^	*U*^12^	*U*^13^	*U*^23^
S1	0.0013 (5)	0.0009 (4)	0.0022 (4)	0.0000 (3)	0.0005 (4)	0.0000 (4)
O3	0.0027 (3)	0.0063 (3)	0.0046 (3)	0.0002 (2)	−0.0001 (2)	−0.0012 (2)
O2	0.0042 (3)	0.0077 (3)	0.0049 (3)	−0.0005 (2)	0.0015 (2)	0.0020 (3)
O4	0.0021 (3)	0.0053 (3)	0.0038 (3)	0.0001 (2)	−0.0003 (2)	0.0001 (2)
O5	0.0062 (3)	0.0039 (3)	0.0037 (3)	−0.0001 (2)	0.0026 (2)	0.0001 (2)
O6	0.0038 (3)	0.0021 (2)	0.0063 (3)	−0.0010 (2)	0.0022 (2)	0.0001 (2)
O7	0.0028 (3)	0.0081 (3)	0.0042 (3)	−0.0004 (2)	0.0007 (2)	0.0004 (2)
H7	0.0104 (8)	0.0207 (11)	0.0168 (10)	0.0000 (8)	0.0047 (7)	−0.0004 (9)
O8	0.0040 (3)	0.0022 (2)	0.0058 (3)	0.0013 (2)	0.0015 (2)	−0.0002 (2)
O9	0.0054 (3)	0.0094 (4)	0.0039 (3)	−0.0011 (3)	0.0016 (2)	0.0003 (3)
O10	0.0035 (3)	0.0072 (3)	0.0050 (3)	0.0005 (2)	0.0014 (2)	−0.0020 (3)
O15	0.0036 (3)	0.0101 (4)	0.0052 (3)	0.0019 (3)	0.0012 (2)	0.0022 (3)
H15	0.0140 (9)	0.0174 (10)	0.0166 (10)	0.0008 (8)	0.0044 (8)	0.0015 (9)
N11	0.00353 (17)	0.00565 (19)	0.00521 (18)	0.00037 (14)	0.00198 (15)	0.00040 (15)
H11A	0.0232 (14)	0.0202 (12)	0.0149 (10)	−0.0037 (11)	0.0025 (10)	−0.0057 (9)
H11B	0.0118 (9)	0.0202 (11)	0.0201 (11)	−0.0011 (8)	0.0082 (8)	0.0021 (10)
H11C	0.0179 (11)	0.0148 (10)	0.0179 (10)	0.0056 (8)	0.0076 (9)	0.0044 (8)
N14	0.00371 (17)	0.00394 (16)	0.00353 (17)	−0.00007 (13)	−0.00001 (13)	−0.00041 (13)
H14A	0.0205 (12)	0.0239 (13)	0.0097 (8)	−0.0023 (10)	0.0022 (8)	−0.0053 (8)
H14B	0.0198 (12)	0.0125 (9)	0.0243 (14)	0.0061 (9)	0.0027 (11)	−0.0024 (9)
H14C	0.0163 (11)	0.0227 (13)	0.0179 (11)	−0.0053 (9)	0.0072 (9)	0.0042 (10)
N21	0.00371 (17)	0.00376 (17)	0.00370 (17)	−0.00009 (13)	0.00047 (14)	0.00026 (13)
H21A	0.0174 (11)	0.0204 (12)	0.0178 (11)	0.0057 (9)	0.0068 (9)	−0.0048 (9)
H21B	0.0191 (12)	0.0146 (10)	0.0218 (13)	−0.0075 (9)	0.0038 (10)	0.0036 (9)
H21C	0.0176 (11)	0.0198 (11)	0.0103 (8)	0.0022 (8)	0.0028 (8)	0.0044 (8)
C15	0.0034 (2)	0.0064 (3)	0.0038 (2)	−0.0001 (2)	0.00083 (19)	−0.0014 (2)
H15A	0.0211 (13)	0.0320 (17)	0.0173 (11)	−0.0101 (12)	0.0081 (11)	0.0032 (12)
H15B	0.0194 (12)	0.0198 (12)	0.0240 (14)	0.0089 (10)	0.0014 (11)	−0.0090 (11)
C16	0.0029 (2)	0.0038 (2)	0.0037 (2)	0.00049 (18)	0.00095 (19)	0.00052 (19)
C17	0.0026 (2)	0.0055 (3)	0.0053 (3)	0.00030 (19)	0.0011 (2)	0.0011 (2)
H17A	0.0146 (10)	0.0279 (15)	0.0150 (10)	−0.0087 (10)	0.0019 (8)	−0.0037 (10)
H17B	0.0212 (13)	0.0136 (10)	0.0333 (18)	0.0086 (9)	0.0131 (13)	0.0081 (11)
C18	0.0022 (2)	0.0031 (2)	0.0033 (2)	0.00014 (17)	0.00010 (18)	−0.00052 (18)
C19	0.0028 (2)	0.0036 (2)	0.0038 (2)	−0.00029 (17)	0.00102 (19)	0.00007 (18)
C20	0.0038 (2)	0.0052 (3)	0.0040 (2)	0.00017 (19)	0.0014 (2)	0.00104 (19)
H20A	0.0187 (12)	0.0167 (11)	0.0230 (14)	−0.0069 (9)	0.0028 (10)	0.0060 (10)
H20B	0.0221 (13)	0.0288 (16)	0.0150 (10)	0.0096 (12)	0.0081 (10)	−0.0033 (11)

### Bis(glycinium) sulfate–glycine (1/1) (20K) Geometric parameters (Å, º)

**Table:**

S1—O5	1.470 (2)	N14—H14B	1.022 (3)
S1—O8	1.472 (2)	N14—H14A	1.028 (3)
S1—O6	1.477 (2)	N14—H14C	1.038 (3)
S1—O4	1.480 (2)	N14—C15	1.4758 (11)
O3—C18	1.2778 (14)	N21—H21B	1.020 (3)
O3—H15^i^	1.408 (4)	N21—H21C	1.031 (3)
O2—C16	1.2181 (15)	N21—H21A	1.039 (3)
O7—H7	1.030 (3)	N21—C20	1.4726 (11)
O7—C19	1.3063 (14)	C15—H15B	1.086 (3)
O9—C19	1.2149 (15)	C15—H15A	1.088 (4)
O10—C18	1.2333 (15)	C15—C18	1.5164 (13)
O15—H15	1.070 (4)	C16—C20	1.5087 (13)
O15—C16	1.2987 (14)	C17—H17A	1.089 (3)
N11—H11A	1.024 (3)	C17—H17B	1.095 (3)
N11—H11B	1.042 (3)	C17—C19	1.5092 (13)
N11—H11C	1.045 (3)	C20—H20A	1.088 (3)
N11—C17	1.4800 (12)	C20—H20B	1.090 (3)
			
O5—S1—O8	110.60 (14)	H15B—C15—H15A	107.9 (4)
O5—S1—O6	109.68 (14)	H15B—C15—N14	109.0 (2)
O8—S1—O6	109.68 (13)	H15A—C15—N14	109.1 (2)
O5—S1—O4	110.31 (13)	H15B—C15—C18	110.2 (2)
O8—S1—O4	108.66 (14)	H15A—C15—C18	109.8 (2)
O6—S1—O4	107.86 (13)	N14—C15—C18	110.68 (7)
C18—O3—H15^i^	117.66 (16)	O2—C16—O15	125.77 (11)
H7—O7—C19	111.1 (2)	O2—C16—C20	122.44 (9)
H15—O15—C16	112.3 (2)	O15—C16—C20	111.79 (9)
H11A—N11—H11B	107.0 (3)	H17A—C17—H17B	108.8 (4)
H11A—N11—H11C	107.2 (3)	H17A—C17—N11	108.7 (2)
H11B—N11—H11C	108.7 (3)	H17B—C17—N11	109.2 (2)
H11A—N11—C17	113.2 (2)	H17A—C17—C19	109.2 (2)
H11B—N11—C17	110.6 (2)	H17B—C17—C19	109.0 (2)
H11C—N11—C17	110.0 (2)	N11—C17—C19	111.83 (7)
H14B—N14—H14A	109.0 (3)	O10—C18—O3	126.16 (11)
H14B—N14—H14C	110.1 (3)	O10—C18—C15	120.14 (9)
H14A—N14—H14C	106.9 (3)	O3—C18—C15	113.70 (9)
H14B—N14—C15	110.3 (2)	O9—C19—O7	124.83 (10)
H14A—N14—C15	111.0 (2)	O9—C19—C17	121.54 (10)
H14C—N14—C15	109.5 (2)	O7—C19—C17	113.62 (9)
H21B—N21—H21C	108.6 (3)	H20A—C20—H20B	107.2 (4)
H21B—N21—H21A	110.1 (3)	H20A—C20—N21	109.3 (2)
H21C—N21—H21A	105.8 (3)	H20B—C20—N21	109.7 (2)
H21B—N21—C20	111.4 (2)	H20A—C20—C16	109.4 (2)
H21C—N21—C20	111.5 (2)	H20B—C20—C16	110.9 (2)
H21A—N21—C20	109.4 (2)	N21—C20—C16	110.28 (7)
			
H14B—N14—C15—H15B	−175.7 (4)	H15A—C15—C18—O3	−60.4 (3)
H14A—N14—C15—H15B	−54.8 (4)	N14—C15—C18—O3	179.09 (9)
H14C—N14—C15—H15B	63.0 (4)	H7—O7—C19—O9	5.4 (3)
H14B—N14—C15—H15A	−58.0 (4)	H7—O7—C19—C17	−174.9 (2)
H14A—N14—C15—H15A	62.9 (4)	H17A—C17—C19—O9	−38.9 (3)
H14C—N14—C15—H15A	−179.3 (4)	H17B—C17—C19—O9	79.8 (3)
H14B—N14—C15—C18	63.0 (3)	N11—C17—C19—O9	−159.24 (11)
H14A—N14—C15—C18	−176.1 (3)	H17A—C17—C19—O7	141.4 (3)
H14C—N14—C15—C18	−58.4 (3)	H17B—C17—C19—O7	−99.8 (3)
H15—O15—C16—O2	−9.2 (3)	N11—C17—C19—O7	21.07 (12)
H15—O15—C16—C20	170.4 (2)	H21B—N21—C20—H20A	−176.3 (4)
H11A—N11—C17—H17A	165.8 (4)	H21C—N21—C20—H20A	62.3 (3)
H11B—N11—C17—H17A	45.7 (3)	H21A—N21—C20—H20A	−54.4 (4)
H11C—N11—C17—H17A	−74.4 (3)	H21B—N21—C20—H20B	66.4 (4)
H11A—N11—C17—H17B	47.1 (4)	H21C—N21—C20—H20B	−55.0 (4)
H11B—N11—C17—H17B	−72.9 (3)	H21A—N21—C20—H20B	−171.6 (4)
H11C—N11—C17—H17B	167.0 (3)	H21B—N21—C20—C16	−56.0 (3)
H11A—N11—C17—C19	−73.6 (3)	H21C—N21—C20—C16	−177.5 (2)
H11B—N11—C17—C19	166.3 (2)	H21A—N21—C20—C16	65.9 (3)
H11C—N11—C17—C19	46.2 (2)	O2—C16—C20—H20A	118.9 (3)
H15^i^—O3—C18—O10	4.8 (2)	O15—C16—C20—H20A	−60.7 (3)
H15^i^—O3—C18—C15	−175.85 (18)	O2—C16—C20—H20B	−123.2 (3)
H15B—C15—C18—O10	−122.1 (3)	O15—C16—C20—H20B	57.2 (3)
H15A—C15—C18—O10	119.1 (3)	O2—C16—C20—N21	−1.38 (14)
N14—C15—C18—O10	−1.47 (14)	O15—C16—C20—N21	179.02 (9)
H15B—C15—C18—O3	58.5 (3)		

Symmetry code: (i) −*x*+3, *y*+1/2, −*z*+2.

### Bis(glycinium) sulfate–glycine (1/1) (20K) Hydrogen-bond geometry (Å, º)

**Table:**

*D*—H···*A*	*D*—H	H···*A*	*D*···*A*	*D*—H···*A*
O7—H7···O4^ii^	1.030 (3)	1.497 (3)	2.5258 (17)	176.3 (4)
O15—H15···O3^iii^	1.070 (3)	1.408 (3)	2.4777 (15)	179.0 (4)
N11—H11*A*···O9^iv^	1.024 (4)	1.894 (4)	2.8099 (13)	147.1 (3)
N11—H11*B*···O6^v^	1.042 (3)	1.715 (3)	2.7557 (14)	176.7 (3)
N11—H11*B*···O8^v^	1.042 (3)	2.522 (4)	3.1097 (14)	115.1 (3)
N11—H11*C*···O3^iv^	1.046 (4)	1.741 (4)	2.7736 (14)	168.5 (3)
N14—H14*A*···O4^ii^	1.028 (3)	2.233 (4)	2.9718 (13)	127.4 (3)
N14—H14*A*···O6^ii^	1.028 (3)	2.026 (4)	2.9771 (13)	152.7 (3)
N14—H14*B*···O8^vi^	1.023 (4)	1.972 (4)	2.8658 (12)	144.4 (3)
N14—H14*B*···O2^vii^	1.023 (4)	2.481 (4)	2.9914 (15)	110.2 (3)
N14—H14*C*···O5^vii^	1.039 (4)	1.815 (4)	2.7952 (13)	155.8 (3)
N21—H21*A*···O5^vii^	1.039 (4)	1.757 (4)	2.7501 (13)	158.5 (3)
N21—H21*B*···O6	1.020 (4)	2.110 (4)	2.8758 (12)	130.3 (3)
N21—H21*B*···O10^viii^	1.020 (4)	2.200 (4)	2.8612 (15)	121.0 (3)
N21—H21*C*···O4^ii^	1.031 (3)	2.313 (4)	2.9677 (13)	120.1 (3)
N21—H21*C*···O8^ii^	1.031 (3)	1.893 (3)	2.9010 (13)	165.1 (3)
C15—H15*A*···O15^ix^	1.088 (5)	2.365 (5)	3.2496 (17)	137.4 (3)
C15—H15*B*···O9	1.085 (4)	2.537 (5)	3.5320 (18)	152.0 (3)
C17—H17*B*···O10^x^	1.095 (4)	2.280 (4)	3.3311 (16)	160.2 (4)
C20—H20*A*···O9	1.087 (4)	2.369 (4)	3.2703 (18)	139.2 (3)
C20—H20*A*···O4^ii^	1.087 (4)	2.532 (4)	3.0948 (15)	111.2 (3)

Symmetry codes: (ii) −*x*+2, *y*+1/2, −*z*; (iii) −*x*+3, *y*−1/2, −*z*+2; (iv) *x*, *y*, *z*−1; (v) −*x*+3, *y*+1/2, −*z*; (vi) *x*, *y*+1, *z*; (vii) −*x*+2, *y*+1/2, −*z*+1; (viii) −*x*+2, *y*−1/2, −*z*+1; (ix) −*x*+3, *y*+1/2, −*z*+1; (x) −*x*+3, *y*−1/2, −*z*+1.

### Bis(glycinium) sulfate–glycine (1/1) (298KModel1) Crystal data

**Table:**

2C_2_H_6_NO_2_^+^·SO_4_^2^^−^·C_2_H_5_NO_2_	*F*(000) = 130.536
*M**_r_* = 323.28	*D*_x_ = 1.693 Mg m^−^^3^
Monoclinic, *P*2_1_	Neutrons radiation, λ = 1 Å
Hall symbol: P2yb	Cell parameters from 3228 reflections
*a* = 9.3910 (14) Å	θ = 6.6–83.3°
*b* = 12.6021 (18) Å	µ = 0.49 mm^−^^1^
*c* = 5.7125 (7) Å	*T* = 298 K
β = 110.306 (13)°	Block, colorless
*V* = 634.04 (16) Å^3^	2.80 × 2.80 × 2.80 mm
*Z* = 2	

### Bis(glycinium) sulfate–glycine (1/1) (298KModel1) Data collection

**Table:**

Time-of-flight Laue-type single crystal neutron diffractometer	10685 reflections with *I* > 2σ(*I*)
Radiation source: spallation neutron	*R*_int_ = N/A
Detector resolution: 4 pixels mm^-1^	θ_max_ = 83.7°, θ_min_ = 7.9°
time–of–flight Laue method scans	*h* = −15→15
14190 measured reflections	*k* = −21→20
3132 independent reflections	*l* = −9→9

### Bis(glycinium) sulfate–glycine (1/1) (298KModel1) Refinement

**Table:**

Refinement on *F*^2^	All H-atom parameters refined
Least-squares matrix: full	*w* = 1/[σ^2^(*F*_o_^2^) + (0.1405*P*)^2^] where *P* = (*F*_o_^2^ + 2*F*_c_^2^)/3
*R*[*F*^2^ > 2σ(*F*^2^)] = 0.080	(Δ/σ)_max_ = 0.001
*wR*(*F*^2^) = 0.209	Δρ_max_ = 3.50 e Å^−^^3^
*S* = 1.05	Δρ_min_ = −6.06 e Å^−^^3^
14190 reflections	Extinction correction: SHELXL-2018/3 (Sheldrick 2018)
357 parameters	Extinction coefficient: 0.119 (3)
1 restraint	Absolute structure: Indeterminate for a neutron structure

### Bis(glycinium) sulfate–glycine (1/1) (298KModel1) Special details

**Table:**

Geometry. All esds (except the esd in the dihedral angle between two l.s. planes) are estimated using the full covariance matrix. The cell esds are taken into account individually in the estimation of esds in distances, angles and torsion angles; correlations between esds in cell parameters are only used when they are defined by crystal symmetry. An approximate (isotropic) treatment of cell esds is used for estimating esds involving l.s. planes.
Refinement. reflns_Friedel_fraction is defined as the number of unique Friedel pairs measured divided by the number that would be possible theoretically, ignoring centric projections and systematic absences.

### Bis(glycinium) sulfate–glycine (1/1) (298KModel1) Fractional atomic coordinates and isotropic or equivalent isotropic displacement parameters (Å^2^)

**Table:**

	*x*	*y*	*z*	*U*_iso_*/*U*_eq_	Occ. (<1)
S1	0.9997 (5)	0.7500 (5)	0.7732 (8)	0.0132 (7)	
O2	0.9154 (4)	0.8459 (2)	0.7922 (8)	0.0239 (7)	
O3	0.5395 (5)	1.4692 (4)	0.1995 (9)	0.0336 (9)	
O4	0.7789 (5)	1.4949 (3)	0.2233 (8)	0.0308 (8)	
O5	1.1424 (3)	0.7495 (4)	0.9937 (5)	0.0239 (5)	
O6	1.0347 (4)	0.7517 (3)	0.5439 (5)	0.0252 (5)	
O7	0.7864 (5)	0.9977 (4)	0.2398 (8)	0.0313 (8)	
O9	0.6071 (4)	1.2431 (4)	1.0767 (7)	0.0318 (7)	
H9	0.7083 (6)	1.2436 (8)	1.0440 (12)	0.0370 (12)	
O10	0.4960 (5)	1.2334 (5)	0.6667 (8)	0.0435 (12)	
O12	0.9126 (5)	0.6560 (3)	0.7868 (8)	0.0260 (7)	
O15	0.5516 (5)	1.0201 (5)	0.2419 (10)	0.0419 (11)	
H15	0.5111 (11)	0.9968 (7)	0.046 (2)	0.050 (2)	
N11	0.3571 (3)	1.2889 (3)	1.1627 (6)	0.0318 (6)	0.874 (8)
H11A	0.4246 (11)	1.3547 (10)	1.200 (2)	0.051 (2)	0.874 (8)
H11B	0.4071 (13)	1.2382 (13)	1.305 (2)	0.063 (3)	0.874 (8)
H11C	0.2518 (10)	1.3094 (9)	1.176 (2)	0.048 (2)	0.874 (8)
N11B	0.364 (2)	1.224 (2)	1.167 (4)	0.0318 (6)	0.126 (8)
H11D	0.427371	1.284076	1.276505	0.051 (2)	0.126 (8)
H11E	0.421468	1.153071	1.217566	0.063 (3)	0.126 (8)
H11F	0.260990	1.218760	1.192923	0.048 (2)	0.126 (8)
N16	0.9163 (3)	1.07320 (14)	0.7057 (4)	0.0229 (4)	
H16A	0.9555 (11)	1.1246 (8)	0.6000 (17)	0.0451 (18)	
H16B	0.9613 (13)	0.9994 (7)	0.7083 (17)	0.053 (2)	
H16C	0.9530 (12)	1.1040 (6)	0.8831 (14)	0.0414 (17)	
N21	0.8957 (3)	1.42963 (15)	0.6984 (4)	0.0221 (4)	
H21A	0.9418 (11)	1.3815 (9)	0.6004 (18)	0.049 (2)	
H21B	0.9277 (12)	1.3988 (7)	0.8734 (14)	0.046 (2)	
H21C	0.9367 (12)	1.5046 (7)	0.7098 (16)	0.052 (2)	
C13	0.6830 (3)	1.4683 (2)	0.3127 (6)	0.0204 (5)	
C14	0.7510 (4)	1.0688 (3)	0.6007 (6)	0.0257 (6)	
H14A	0.7068 (12)	1.1483 (8)	0.597 (2)	0.059 (3)	
H14B	0.7088 (15)	1.0190 (11)	0.719 (2)	0.064 (3)	
C17	0.3401 (3)	1.2452 (3)	0.9149 (5)	0.0256 (5)	
H17A	0.2645 (11)	1.2958 (10)	0.7802 (18)	0.061 (3)	
H17B	0.2911 (14)	1.1675 (8)	0.895 (3)	0.073 (4)	
C18	0.6979 (4)	1.0246 (2)	0.3395 (6)	0.0230 (6)	
C19	0.4893 (3)	1.2402 (2)	0.8730 (5)	0.0221 (5)	
C20	0.7303 (4)	1.4306 (3)	0.5794 (6)	0.0258 (6)	
H20A	0.6812 (15)	1.4810 (13)	0.682 (2)	0.074 (4)	
H20B	0.6890 (13)	1.3519 (10)	0.584 (2)	0.065 (3)	

### Bis(glycinium) sulfate–glycine (1/1) (298KModel1) Atomic displacement parameters (Å^2^)

**Table:**

	*U*^11^	*U*^22^	*U*^33^	*U*^12^	*U*^13^	*U*^23^
S1	0.0134 (18)	0.0106 (13)	0.0159 (17)	0.0002 (19)	0.0057 (14)	−0.0022 (19)
O2	0.0244 (15)	0.0152 (12)	0.0353 (19)	0.0016 (11)	0.0145 (14)	0.0063 (10)
O3	0.0181 (15)	0.0438 (19)	0.0325 (19)	0.0056 (14)	0.0009 (13)	0.0029 (15)
O4	0.0239 (17)	0.0405 (18)	0.0244 (16)	0.0118 (14)	0.0039 (13)	−0.0039 (14)
O5	0.0147 (11)	0.0363 (12)	0.0188 (10)	0.0010 (15)	0.0035 (9)	0.0018 (14)
O6	0.0337 (15)	0.0271 (11)	0.0178 (10)	−0.0003 (13)	0.0127 (10)	−0.0006 (15)
O7	0.0252 (17)	0.0418 (18)	0.0255 (16)	−0.0112 (15)	0.0069 (14)	0.0043 (14)
O9	0.0142 (12)	0.0518 (19)	0.0286 (14)	−0.0027 (18)	0.0065 (11)	0.0003 (16)
H9	0.021 (2)	0.050 (3)	0.039 (3)	−0.004 (3)	0.010 (2)	0.002 (3)
O10	0.0310 (19)	0.071 (3)	0.0268 (16)	−0.0066 (19)	0.0084 (14)	0.006 (2)
O12	0.0274 (17)	0.0159 (12)	0.035 (2)	0.0001 (11)	0.0111 (14)	−0.0072 (11)
O15	0.0222 (18)	0.063 (3)	0.039 (2)	−0.014 (2)	0.0087 (17)	−0.0124 (19)
H15	0.031 (3)	0.044 (3)	0.071 (7)	−0.002 (4)	0.015 (4)	−0.003 (3)
N11	0.0174 (10)	0.0492 (17)	0.0316 (11)	−0.0032 (12)	0.0119 (9)	−0.0065 (11)
H11A	0.037 (4)	0.074 (6)	0.048 (5)	−0.025 (5)	0.020 (4)	−0.017 (4)
H11B	0.047 (5)	0.088 (9)	0.051 (5)	0.025 (6)	0.014 (4)	−0.009 (6)
H11C	0.028 (4)	0.060 (5)	0.061 (6)	−0.010 (4)	0.022 (4)	−0.005 (3)
N11B	0.0174 (10)	0.0492 (17)	0.0316 (11)	−0.0032 (12)	0.0119 (9)	−0.0065 (11)
H11D	0.037 (4)	0.074 (6)	0.048 (5)	−0.025 (5)	0.020 (4)	−0.017 (4)
H11E	0.047 (5)	0.088 (9)	0.051 (5)	0.025 (6)	0.014 (4)	−0.009 (6)
H11F	0.028 (4)	0.060 (5)	0.061 (6)	−0.010 (4)	0.022 (4)	−0.005 (3)
N16	0.0270 (10)	0.0200 (8)	0.0179 (8)	−0.0019 (6)	0.0033 (7)	0.0037 (7)
H16A	0.039 (4)	0.062 (5)	0.033 (4)	0.002 (3)	0.012 (3)	−0.012 (3)
H16B	0.057 (5)	0.044 (4)	0.042 (4)	−0.010 (3)	−0.003 (4)	0.026 (4)
H16C	0.056 (5)	0.035 (3)	0.028 (3)	−0.010 (2)	0.008 (3)	−0.004 (3)
N21	0.0244 (9)	0.0216 (8)	0.0161 (8)	0.0036 (6)	0.0017 (7)	−0.0029 (7)
H21A	0.040 (4)	0.070 (5)	0.037 (4)	−0.004 (3)	0.011 (3)	0.015 (4)
H21B	0.063 (6)	0.042 (3)	0.027 (3)	0.010 (3)	0.007 (3)	0.003 (3)
H21C	0.057 (5)	0.046 (4)	0.040 (4)	0.009 (3)	−0.001 (4)	−0.026 (4)
C13	0.0177 (11)	0.0186 (10)	0.0218 (12)	0.0033 (8)	0.0032 (9)	0.0015 (9)
C14	0.0268 (14)	0.0270 (12)	0.0252 (13)	−0.0068 (10)	0.0113 (11)	−0.0012 (10)
H14A	0.045 (5)	0.052 (4)	0.069 (7)	−0.027 (4)	0.005 (4)	0.017 (4)
H14B	0.071 (7)	0.088 (7)	0.043 (5)	−0.003 (4)	0.034 (5)	−0.028 (6)
C17	0.0144 (10)	0.0276 (10)	0.0322 (12)	−0.0032 (12)	0.0048 (9)	−0.0006 (11)
H17A	0.039 (4)	0.095 (7)	0.047 (4)	0.010 (4)	0.010 (4)	0.030 (5)
H17B	0.058 (6)	0.045 (4)	0.127 (11)	−0.032 (6)	0.046 (7)	−0.025 (4)
C18	0.0217 (12)	0.0226 (12)	0.0244 (13)	−0.0035 (9)	0.0076 (10)	−0.0026 (9)
C19	0.0170 (10)	0.0243 (11)	0.0246 (10)	−0.0007 (9)	0.0068 (8)	0.0022 (9)
C20	0.0215 (13)	0.0313 (13)	0.0246 (14)	0.0060 (10)	0.0079 (11)	−0.0011 (11)
H20A	0.059 (6)	0.120 (10)	0.049 (6)	−0.001 (6)	0.027 (5)	0.035 (7)
H20B	0.055 (6)	0.064 (5)	0.059 (6)	0.029 (4)	−0.003 (4)	−0.031 (5)

### Bis(glycinium) sulfate–glycine (1/1) (298KModel1) Geometric parameters (Å, º)

**Table:**

S1—O6	1.456 (5)	N11B—H11D	1.0300
S1—O12	1.457 (6)	N11B—C17	1.41 (2)
S1—O2	1.470 (6)	N16—H16B	1.020 (8)
S1—O5	1.487 (5)	N16—H16C	1.026 (7)
O3—C13	1.276 (5)	N16—H16A	1.036 (9)
O3—H15^i^	1.361 (12)	N16—C14	1.458 (4)
O4—C13	1.227 (5)	N21—H21C	1.014 (8)
O7—C18	1.208 (5)	N21—H21B	1.016 (7)
O9—H9	1.030 (7)	N21—H21A	1.018 (10)
O9—C19	1.298 (4)	N21—C20	1.464 (4)
O10—C19	1.205 (5)	C13—C20	1.508 (4)
O15—H15	1.089 (12)	C14—H14A	1.082 (9)
O15—C18	1.292 (5)	C14—H14B	1.093 (11)
N11—H11B	1.010 (12)	C14—C18	1.507 (5)
N11—H11A	1.021 (10)	C17—H17A	1.060 (10)
N11—H11C	1.049 (9)	C17—H17B	1.071 (9)
N11—C17	1.475 (4)	C17—C19	1.503 (4)
N11B—H11E	1.0300	C20—H20B	1.068 (10)
N11B—H11F	1.0300	C20—H20A	1.072 (12)
			
O6—S1—O12	111.5 (4)	H21A—N21—C20	109.6 (6)
O6—S1—O2	110.5 (4)	O4—C13—O3	125.8 (4)
O12—S1—O2	109.7 (3)	O4—C13—C20	120.3 (3)
O6—S1—O5	110.1 (3)	O3—C13—C20	113.8 (3)
O12—S1—O5	108.0 (4)	H14A—C14—H14B	108.9 (12)
O2—S1—O5	106.9 (4)	H14A—C14—N16	108.6 (7)
C13—O3—H15^i^	117.0 (5)	H14B—C14—N16	109.3 (8)
H9—O9—C19	113.0 (5)	H14A—C14—C18	109.4 (7)
H15—O15—C18	113.3 (6)	H14B—C14—C18	109.5 (7)
H11B—N11—H11A	105.2 (11)	N16—C14—C18	111.2 (3)
H11B—N11—H11C	106.6 (10)	H17A—C17—H17B	108.5 (12)
H11A—N11—H11C	108.5 (8)	H17A—C17—N11B	133.5 (12)
H11B—N11—C17	113.6 (10)	H17B—C17—N11B	81.1 (13)
H11A—N11—C17	110.9 (6)	H17A—C17—N11	107.1 (7)
H11C—N11—C17	111.6 (6)	H17B—C17—N11	110.1 (9)
H11E—N11B—H11F	109.5	H17A—C17—C19	109.6 (7)
H11E—N11B—H11D	109.5	H17B—C17—C19	109.6 (7)
H11F—N11B—H11D	109.5	N11B—C17—C19	109.2 (8)
H11E—N11B—C17	109.5	N11—C17—C19	111.8 (2)
H11F—N11B—C17	109.5	O7—C18—O15	126.0 (4)
H11D—N11B—C17	109.5	O7—C18—C14	121.7 (3)
H16B—N16—H16C	109.6 (7)	O15—C18—C14	112.4 (4)
H16B—N16—H16A	110.5 (10)	O10—C19—O9	124.1 (3)
H16C—N16—H16A	105.9 (7)	O10—C19—C17	121.8 (3)
H16B—N16—C14	110.4 (7)	O9—C19—C17	114.1 (3)
H16C—N16—C14	111.4 (6)	H20B—C20—H20A	108.0 (13)
H16A—N16—C14	109.1 (6)	H20B—C20—N21	108.5 (7)
H21C—N21—H21B	108.5 (7)	H20A—C20—N21	109.8 (8)
H21C—N21—H21A	111.4 (10)	H20B—C20—C13	109.6 (7)
H21B—N21—H21A	106.1 (8)	H20A—C20—C13	109.5 (8)
H21C—N21—C20	109.9 (7)	N21—C20—C13	111.4 (3)
H21B—N21—C20	111.3 (7)		
			
H15^i^—O3—C13—O4	3.7 (7)	H14A—C14—C18—O7	119.8 (9)
H15^i^—O3—C13—C20	−176.4 (5)	H14B—C14—C18—O7	−121.0 (9)
H16B—N16—C14—H14A	−179.0 (11)	N16—C14—C18—O7	−0.1 (5)
H16C—N16—C14—H14A	59.0 (10)	H14A—C14—C18—O15	−59.9 (9)
H16A—N16—C14—H14A	−57.5 (11)	H14B—C14—C18—O15	59.3 (9)
H16B—N16—C14—H14B	62.4 (11)	N16—C14—C18—O15	−179.8 (4)
H16C—N16—C14—H14B	−59.6 (9)	H9—O9—C19—O10	3.6 (9)
H16A—N16—C14—H14B	−176.1 (9)	H9—O9—C19—C17	−176.9 (7)
H16B—N16—C14—C18	−58.6 (8)	H17A—C17—C19—O10	−42.0 (9)
H16C—N16—C14—C18	179.4 (5)	H17B—C17—C19—O10	77.0 (11)
H16A—N16—C14—C18	62.9 (6)	N11B—C17—C19—O10	164.1 (12)
H11E—N11B—C17—H17A	161.2	N11—C17—C19—O10	−160.6 (4)
H11F—N11B—C17—H17A	41.2	H17A—C17—C19—O9	138.5 (8)
H11D—N11B—C17—H17A	−78.8	H17B—C17—C19—O9	−102.5 (10)
H11E—N11B—C17—H17B	54.1	N11B—C17—C19—O9	−15.4 (12)
H11F—N11B—C17—H17B	−65.9	N11—C17—C19—O9	19.9 (5)
H11D—N11B—C17—H17B	174.1	H21C—N21—C20—H20B	−175.1 (11)
H11E—N11B—C17—C19	−53.6	H21B—N21—C20—H20B	−54.9 (10)
H11F—N11B—C17—C19	−173.6	H21A—N21—C20—H20B	62.1 (11)
H11D—N11B—C17—C19	66.4	H21C—N21—C20—H20A	−57.3 (12)
H11B—N11—C17—H17A	166.7 (11)	H21B—N21—C20—H20A	62.9 (11)
H11A—N11—C17—H17A	−75.0 (11)	H21A—N21—C20—H20A	179.9 (11)
H11C—N11—C17—H17A	46.1 (10)	H21C—N21—C20—C13	64.2 (7)
H11B—N11—C17—H17B	48.9 (12)	H21B—N21—C20—C13	−175.6 (6)
H11A—N11—C17—H17B	167.2 (11)	H21A—N21—C20—C13	−58.6 (8)
H11C—N11—C17—H17B	−71.7 (11)	O4—C13—C20—H20B	−121.0 (10)
H11B—N11—C17—C19	−73.1 (8)	O3—C13—C20—H20B	59.1 (10)
H11A—N11—C17—C19	45.2 (8)	O4—C13—C20—H20A	120.7 (10)
H11C—N11—C17—C19	166.3 (7)	O3—C13—C20—H20A	−59.2 (10)
H15—O15—C18—O7	−6.2 (10)	O4—C13—C20—N21	−1.0 (5)
H15—O15—C18—C14	173.5 (7)	O3—C13—C20—N21	179.1 (3)

Symmetry code: (i) −*x*+1, *y*+1/2, −*z*.

### Bis(glycinium) sulfate–glycine (1/1) (298KModel1) Hydrogen-bond geometry (Å, º)

**Table:**

*D*—H···*A*	*D*—H	H···*A*	*D*···*A*	*D*—H···*A*
O9—H9···O5^ii^	1.030 (7)	1.492 (7)	2.522 (5)	176.8 (8)
O15—H15···O3^iii^	1.090 (12)	1.361 (12)	2.450 (7)	179.2 (10)
N11—H11*A*···O3^iv^	1.020 (13)	1.802 (13)	2.809 (6)	168.3 (10)
N11—H11*B*···O10^iv^	1.012 (14)	1.942 (12)	2.806 (6)	141.5 (13)
N11—H11*C*···O2^v^	1.050 (11)	1.705 (11)	2.755 (5)	177.7 (8)
N11—H11*C*···O12^v^	1.050 (11)	2.529 (12)	3.132 (6)	115.8 (8)
N16—H16*A*···O6^vi^	1.036 (10)	1.817 (11)	2.786 (4)	154.0 (9)
N16—H16*B*···O2	1.020 (10)	2.074 (10)	2.907 (3)	137.4 (10)
N16—H16*B*···O4^vii^	1.020 (10)	2.334 (14)	2.920 (6)	115.4 (8)
N16—H16*C*···O5^ii^	1.027 (8)	2.258 (10)	2.974 (5)	125.5 (7)
N16—H16*C*···O12^ii^	1.027 (8)	1.982 (9)	2.974 (5)	161.7 (10)
N21—H21*A*···O6^vi^	1.018 (11)	1.879 (12)	2.828 (4)	153.8 (10)
N21—H21*B*···O2^ii^	1.016 (8)	2.076 (9)	3.024 (5)	154.3 (10)
N21—H21*B*···O5^ii^	1.016 (8)	2.213 (10)	2.968 (5)	129.9 (7)
N21—H21*C*···O12^viii^	1.014 (9)	1.989 (10)	2.892 (4)	147.0 (10)
N21—H21*C*···O7^vi^	1.014 (9)	2.517 (13)	3.007 (6)	109.2 (7)
C14—H14*A*···O10	1.082 (11)	2.403 (13)	3.287 (7)	138.0 (9)
C17—H17*A*···O7^ix^	1.060 (11)	2.584 (14)	3.404 (6)	133.7 (8)
C17—H17*B*···O4^x^	1.071 (11)	2.305 (12)	3.348 (5)	164.2 (12)
C20—H20*A*···O15^ix^	1.071 (15)	2.423 (15)	3.346 (7)	143.7 (11)
C20—H20*B*···O10	1.069 (13)	2.517 (14)	3.468 (7)	147.9 (10)

Symmetry codes: (ii) −*x*+2, *y*+1/2, −*z*+2; (iii) −*x*+1, *y*−1/2, −*z*; (iv) *x*, *y*, *z*+1; (v) −*x*+1, *y*+1/2, −*z*+2; (vi) −*x*+2, *y*+1/2, −*z*+1; (vii) −*x*+2, *y*−1/2, −*z*+1; (viii) *x*, *y*+1, *z*; (ix) −*x*+1, *y*+1/2, −*z*+1; (x) −*x*+1, *y*−1/2, −*z*+1.

### Bis(glycinium) sulfate–glycine (1/1) (298KModel2) Crystal data

**Table:**

2C_2_H_6_NO_2_^+^·SO_4_^2^^−^·C_2_H_5_NO_2_	*F*(000) = 130.536
*M**_r_* = 323.28	*D*_x_ = 1.693 Mg m^−^^3^
Monoclinic, *P*2_1_	Neutrons radiation, λ = 1 Å
Hall symbol: P2yb	Cell parameters from 3228 reflections
*a* = 9.3910 (14) Å	θ = 6.6–83.3°
*b* = 12.6021 (18) Å	µ = 0.49 mm^−^^1^
*c* = 5.7125 (7) Å	*T* = 298 K
β = 110.306 (13)°	Block, colorless
*V* = 634.04 (16) Å^3^	2.80 × 2.80 × 2.80 mm
*Z* = 2	

### Bis(glycinium) sulfate–glycine (1/1) (298KModel2) Data collection

**Table:**

Time-of-flight Laue-type single crystal neutron diffractometer	10685 reflections with *I* > 2σ(*I*)
Radiation source: spallation neutron	*R*_int_ = N/A
Detector resolution: 4 pixels mm^-1^	θ_max_ = 83.7°, θ_min_ = 7.9°
time–of–flight Laue method scans	*h* = −15→15
14190 measured reflections	*k* = −21→20
3132 independent reflections	*l* = −9→9

### Bis(glycinium) sulfate–glycine (1/1) (298KModel2) Refinement

**Table:**

Refinement on *F*^2^	All H-atom parameters refined
Least-squares matrix: full	*w* = 1/[σ^2^(*F*_o_^2^) + (0.1405*P*)^2^] where *P* = (*F*_o_^2^ + 2*F*_c_^2^)/3
*R*[*F*^2^ > 2σ(*F*^2^)] = 0.080	(Δ/σ)_max_ < 0.001
*wR*(*F*^2^) = 0.209	Δρ_max_ = 1.50 e Å^−^^3^
*S* = 1.05	Δρ_min_ = −1.70 e Å^−^^3^
14190 reflections	Extinction correction: SHELXL-2018/3 (Sheldrick 2015)
358 parameters	Extinction coefficient: 0.106 (5)
8 restraints	Absolute structure: Indeterminate for a neutron structure

### Bis(glycinium) sulfate–glycine (1/1) (298KModel2) Special details

**Table:**

Geometry. All esds (except the esd in the dihedral angle between two l.s. planes) are estimated using the full covariance matrix. The cell esds are taken into account individually in the estimation of esds in distances, angles and torsion angles; correlations between esds in cell parameters are only used when they are defined by crystal symmetry. An approximate (isotropic) treatment of cell esds is used for estimating esds involving l.s. planes.
Refinement. reflns_Friedel_fraction is defined as the number of unique Friedel pairs measured divided by the number that would be possible theoretically, ignoring centric projections and systematic absences.

### Bis(glycinium) sulfate–glycine (1/1) (298KModel2) Fractional atomic coordinates and isotropic or equivalent isotropic displacement parameters (Å^2^)

**Table:**

	*x*	*y*	*z*	*U*_iso_*/*U*_eq_	Occ. (<1)
S1	0.9997 (5)	0.7502 (5)	0.7732 (8)	0.0131 (7)	
O2	0.9154 (4)	0.8462 (2)	0.7921 (8)	0.0238 (7)	
O3	0.5395 (5)	1.4695 (4)	0.1996 (9)	0.0337 (9)	
H3	0.5153 (11)	1.4925 (10)	0.011 (8)	0.046 (2)	0.124 (8)
O4	0.7790 (5)	1.4951 (3)	0.2233 (8)	0.0307 (8)	
O5	1.1424 (3)	0.7497 (4)	0.9937 (5)	0.0239 (5)	
O6	1.0347 (4)	0.7519 (3)	0.5440 (5)	0.0252 (5)	
O7	0.7864 (5)	0.9980 (3)	0.2398 (8)	0.0313 (8)	
O9	0.6071 (4)	1.2434 (4)	1.0767 (7)	0.0317 (7)	
H9	0.7082 (6)	1.2438 (8)	1.0440 (12)	0.0369 (12)	
O10	0.4960 (5)	1.2336 (5)	0.6668 (8)	0.0434 (12)	
O12	0.9126 (5)	0.6563 (3)	0.7868 (8)	0.0261 (7)	
O15	0.5517 (5)	1.0205 (5)	0.2419 (10)	0.0419 (11)	
H15	0.5131 (11)	0.9970 (8)	0.051 (2)	0.046 (2)	0.876 (8)
N11	0.3571 (3)	1.2892 (3)	1.1626 (6)	0.0316 (7)	0.876 (8)
H11A	0.4246 (12)	1.3552 (10)	1.200 (2)	0.052 (2)	0.876 (8)
H11B	0.4077 (13)	1.2388 (13)	1.305 (2)	0.062 (3)	0.876 (8)
H11C	0.2518 (10)	1.3095 (9)	1.176 (2)	0.048 (2)	0.876 (8)
N11B	0.365 (2)	1.226 (2)	1.167 (5)	0.0316 (7)	0.124 (8)
H11D	0.426075	1.156788	1.221059	0.052 (2)	0.124 (8)
H11E	0.262414	1.218805	1.193317	0.062 (3)	0.124 (8)
H11F	0.425392	1.288121	1.273121	0.048 (2)	0.124 (8)
N16	0.9164 (3)	1.07348 (15)	0.7057 (4)	0.0228 (4)	
H16A	0.9553 (11)	1.1250 (8)	0.5999 (17)	0.0454 (18)	
H16B	0.9613 (13)	0.9997 (7)	0.7084 (17)	0.053 (2)	
H16C	0.9530 (12)	1.1042 (6)	0.8830 (14)	0.0414 (17)	
N21	0.8957 (3)	1.42989 (15)	0.6984 (4)	0.0220 (4)	
H21A	0.9420 (11)	1.3818 (9)	0.6005 (18)	0.049 (2)	
H21B	0.9277 (12)	1.3990 (7)	0.8735 (14)	0.046 (2)	
H21C	0.9368 (12)	1.5049 (7)	0.7098 (16)	0.053 (2)	
C13	0.6830 (3)	1.4686 (2)	0.3128 (6)	0.0203 (5)	
C14	0.7510 (4)	1.0691 (3)	0.6007 (6)	0.0257 (6)	
H14A	0.7067 (12)	1.1486 (8)	0.597 (2)	0.060 (3)	
H14B	0.7088 (15)	1.0192 (11)	0.719 (2)	0.064 (3)	
C17	0.3401 (3)	1.2455 (3)	0.9149 (5)	0.0256 (5)	
H17A	0.2643 (11)	1.2962 (10)	0.7802 (18)	0.062 (3)	
H17B	0.2910 (14)	1.1679 (8)	0.895 (3)	0.073 (4)	
C18	0.6979 (4)	1.0248 (2)	0.3394 (6)	0.0230 (6)	
C19	0.4894 (3)	1.2405 (2)	0.8731 (5)	0.0221 (5)	
C20	0.7302 (4)	1.4308 (3)	0.5793 (6)	0.0258 (6)	
H20A	0.6814 (15)	1.4815 (13)	0.683 (2)	0.074 (4)	
H20B	0.6892 (13)	1.3521 (10)	0.584 (2)	0.066 (3)	

### Bis(glycinium) sulfate–glycine (1/1) (298KModel2) Atomic displacement parameters (Å^2^)

**Table:**

	*U*^11^	*U*^22^	*U*^33^	*U*^12^	*U*^13^	*U*^23^
S1	0.0133 (18)	0.0105 (13)	0.0159 (17)	−0.0021 (19)	0.0057 (14)	0.0003 (19)
O2	0.0243 (15)	0.0152 (12)	0.0352 (19)	0.0063 (10)	0.0144 (14)	0.0017 (11)
O3	0.0179 (15)	0.0440 (19)	0.0327 (19)	0.0028 (15)	0.0008 (13)	0.0059 (15)
H3	0.026 (3)	0.044 (3)	0.062 (7)	−0.005 (3)	0.008 (4)	−0.004 (4)
O4	0.0238 (17)	0.0403 (18)	0.0245 (17)	−0.0039 (14)	0.0041 (13)	0.0119 (14)
O5	0.0147 (11)	0.0363 (12)	0.0188 (10)	0.0019 (14)	0.0036 (9)	0.0011 (15)
O6	0.0337 (15)	0.0270 (11)	0.0179 (10)	−0.0006 (15)	0.0128 (10)	−0.0003 (13)
O7	0.0252 (17)	0.0418 (18)	0.0253 (16)	0.0043 (14)	0.0067 (14)	−0.0111 (15)
O9	0.0142 (12)	0.0517 (19)	0.0287 (14)	0.0003 (16)	0.0067 (11)	−0.0028 (18)
H9	0.021 (2)	0.050 (3)	0.039 (3)	0.002 (3)	0.010 (2)	−0.004 (3)
O10	0.0309 (19)	0.071 (3)	0.0268 (16)	0.005 (2)	0.0083 (14)	−0.0069 (19)
O12	0.0276 (17)	0.0158 (12)	0.035 (2)	−0.0072 (11)	0.0113 (15)	0.0001 (11)
O15	0.0222 (18)	0.064 (3)	0.038 (2)	−0.0124 (19)	0.0085 (17)	−0.014 (2)
H15	0.026 (3)	0.044 (3)	0.062 (7)	−0.005 (3)	0.008 (4)	−0.004 (4)
N11	0.0176 (10)	0.0485 (18)	0.0314 (11)	−0.0062 (11)	0.0119 (9)	−0.0033 (12)
H11A	0.038 (4)	0.075 (6)	0.048 (5)	−0.017 (4)	0.020 (4)	−0.024 (5)
H11B	0.046 (5)	0.088 (9)	0.048 (5)	−0.009 (6)	0.013 (4)	0.023 (6)
H11C	0.028 (4)	0.059 (5)	0.061 (6)	−0.006 (3)	0.023 (4)	−0.011 (4)
N11B	0.0176 (10)	0.0485 (18)	0.0314 (11)	−0.0062 (11)	0.0119 (9)	−0.0033 (12)
H11D	0.038 (4)	0.075 (6)	0.048 (5)	−0.017 (4)	0.020 (4)	−0.024 (5)
H11E	0.046 (5)	0.088 (9)	0.048 (5)	−0.009 (6)	0.013 (4)	0.023 (6)
H11F	0.028 (4)	0.059 (5)	0.061 (6)	−0.006 (3)	0.023 (4)	−0.011 (4)
N16	0.0269 (10)	0.0201 (8)	0.0179 (8)	0.0037 (7)	0.0033 (7)	−0.0019 (6)
H16A	0.039 (4)	0.063 (5)	0.033 (4)	−0.012 (4)	0.011 (3)	0.002 (3)
H16B	0.057 (5)	0.044 (4)	0.042 (4)	0.025 (4)	−0.003 (4)	−0.010 (3)
H16C	0.056 (5)	0.035 (3)	0.028 (3)	−0.004 (3)	0.008 (3)	−0.010 (2)
N21	0.0244 (9)	0.0214 (8)	0.0160 (8)	−0.0029 (7)	0.0016 (7)	0.0036 (6)
H21A	0.040 (4)	0.069 (5)	0.036 (4)	0.015 (4)	0.011 (3)	−0.003 (3)
H21B	0.063 (6)	0.042 (3)	0.026 (3)	0.003 (3)	0.007 (3)	0.010 (3)
H21C	0.057 (5)	0.045 (4)	0.041 (4)	−0.026 (4)	−0.001 (4)	0.009 (3)
C13	0.0177 (11)	0.0186 (10)	0.0218 (12)	0.0014 (9)	0.0032 (9)	0.0032 (8)
C14	0.0267 (14)	0.0270 (12)	0.0251 (13)	−0.0012 (10)	0.0113 (11)	−0.0067 (10)
H14A	0.045 (5)	0.053 (4)	0.069 (7)	0.017 (4)	0.005 (4)	−0.027 (4)
H14B	0.072 (7)	0.089 (7)	0.044 (5)	−0.028 (6)	0.034 (5)	−0.003 (4)
C17	0.0143 (10)	0.0276 (10)	0.0324 (12)	−0.0007 (11)	0.0048 (9)	−0.0031 (12)
H17A	0.040 (4)	0.095 (7)	0.046 (4)	0.031 (5)	0.010 (4)	0.010 (5)
H17B	0.058 (6)	0.045 (4)	0.126 (11)	−0.024 (4)	0.045 (7)	−0.032 (6)
C18	0.0217 (12)	0.0226 (12)	0.0244 (13)	−0.0026 (9)	0.0077 (10)	−0.0035 (9)
C19	0.0170 (10)	0.0243 (11)	0.0246 (11)	0.0021 (9)	0.0068 (8)	−0.0007 (9)
C20	0.0215 (13)	0.0312 (13)	0.0245 (14)	−0.0011 (11)	0.0078 (11)	0.0060 (10)
H20A	0.060 (6)	0.118 (10)	0.049 (6)	0.035 (7)	0.028 (5)	−0.001 (6)
H20B	0.055 (6)	0.064 (5)	0.059 (6)	−0.031 (5)	−0.003 (4)	0.029 (4)

### Bis(glycinium) sulfate–glycine (1/1) (298KModel2) Geometric parameters (Å, º)

**Table:**

S1—O12	1.456 (6)	N11B—H11F	1.0300
S1—O6	1.457 (5)	N11B—H11D	1.0300
S1—O2	1.469 (6)	N11B—C17	1.40 (2)
S1—O5	1.487 (5)	N16—H16B	1.019 (8)
O3—H3	1.06 (4)	N16—H16C	1.026 (7)
O3—C13	1.277 (5)	N16—H16A	1.036 (9)
O3—H15^i^	1.386 (12)	N16—C14	1.459 (4)
H3—H15^i^	0.37 (4)	N21—H21C	1.014 (8)
O4—C13	1.226 (5)	N21—H21B	1.016 (7)
O7—C18	1.208 (5)	N21—H21A	1.019 (10)
O9—H9	1.029 (7)	N21—C20	1.464 (4)
O9—C19	1.298 (4)	C13—C20	1.508 (4)
O10—C19	1.204 (5)	C14—H14A	1.082 (9)
O15—H15	1.066 (12)	C14—H14B	1.093 (11)
O15—C18	1.291 (5)	C14—C18	1.507 (5)
N11—H11B	1.010 (12)	C17—H17A	1.061 (10)
N11—H11A	1.022 (11)	C17—H17B	1.070 (9)
N11—H11C	1.050 (9)	C17—C19	1.503 (4)
N11—C17	1.474 (4)	C20—H20B	1.068 (10)
N11B—H11E	1.0300	C20—H20A	1.074 (12)
			
O12—S1—O6	111.5 (4)	H21B—N21—H21A	106.0 (8)
O12—S1—O2	109.8 (3)	H21C—N21—C20	110.0 (7)
O6—S1—O2	110.4 (4)	H21B—N21—C20	111.3 (7)
O12—S1—O5	108.0 (4)	H21A—N21—C20	109.6 (6)
O6—S1—O5	110.1 (3)	O4—C13—O3	125.8 (4)
O2—S1—O5	107.0 (4)	O4—C13—C20	120.4 (3)
H3—O3—C13	109.5	O3—C13—C20	113.7 (3)
H3—O3—H15^i^	8.0	H14A—C14—H14B	108.9 (12)
C13—O3—H15^i^	117.4 (6)	H14A—C14—N16	108.7 (7)
H15^i^—H3—O3	149 (4)	H14B—C14—N16	109.3 (8)
H9—O9—C19	113.0 (5)	H14A—C14—C18	109.3 (7)
H15—O15—C18	112.7 (7)	H14B—C14—C18	109.5 (7)
H15—O15—H3^ii^	6 (3)	N16—C14—C18	111.1 (3)
C18—O15—H3^ii^	118.9 (11)	H17A—C17—H17B	108.4 (12)
H3^ii^—H15—O15	155.4	H17A—C17—N11B	133.1 (12)
H11B—N11—H11A	105.0 (11)	H17B—C17—N11B	82.1 (14)
H11B—N11—H11C	106.7 (10)	H17A—C17—N11	107.1 (7)
H11A—N11—H11C	108.5 (9)	H17B—C17—N11	110.1 (9)
H11B—N11—C17	113.7 (10)	H17A—C17—C19	109.7 (7)
H11A—N11—C17	110.9 (6)	H17B—C17—C19	109.6 (7)
H11C—N11—C17	111.7 (6)	N11B—C17—C19	108.9 (8)
H11E—N11B—H11F	109.5	N11—C17—C19	111.8 (2)
H11E—N11B—H11D	109.5	O7—C18—O15	126.0 (4)
H11F—N11B—H11D	109.5	O7—C18—C14	121.7 (3)
H11E—N11B—C17	109.5	O15—C18—C14	112.3 (4)
H11F—N11B—C17	109.5	O10—C19—O9	124.2 (3)
H11D—N11B—C17	109.5	O10—C19—C17	121.7 (3)
H16B—N16—H16C	109.5 (7)	O9—C19—C17	114.1 (3)
H16B—N16—H16A	110.6 (10)	H20B—C20—H20A	108.2 (13)
H16C—N16—H16A	105.9 (7)	H20B—C20—N21	108.3 (7)
H16B—N16—C14	110.3 (7)	H20A—C20—N21	109.6 (8)
H16C—N16—C14	111.4 (6)	H20B—C20—C13	109.7 (7)
H16A—N16—C14	109.0 (6)	H20A—C20—C13	109.6 (8)
H21C—N21—H21B	108.5 (7)	N21—C20—C13	111.4 (3)
H21C—N21—H21A	111.3 (10)		
			
C13—O3—H3—H15^i^	176.0	H3^ii^—O15—C18—O7	−6.9
C18—O15—H15—H3^ii^	−169.8	H15—O15—C18—C14	173.9 (7)
H3—O3—C13—O4	3.4	H3^ii^—O15—C18—C14	172.7
H15^i^—O3—C13—O4	4.0 (8)	H14A—C14—C18—O7	119.8 (9)
H3—O3—C13—C20	−176.7	H14B—C14—C18—O7	−120.9 (10)
H15^i^—O3—C13—C20	−176.1 (6)	N16—C14—C18—O7	−0.1 (5)
H16B—N16—C14—H14A	−178.9 (11)	H14A—C14—C18—O15	−59.8 (9)
H16C—N16—C14—H14A	59.2 (10)	H14B—C14—C18—O15	59.4 (9)
H16A—N16—C14—H14A	−57.3 (11)	N16—C14—C18—O15	−179.8 (4)
H16B—N16—C14—H14B	62.3 (11)	H9—O9—C19—O10	3.6 (9)
H16C—N16—C14—H14B	−59.6 (9)	H9—O9—C19—C17	−177.0 (7)
H16A—N16—C14—H14B	−176.1 (10)	H17A—C17—C19—O10	−42.0 (9)
H16B—N16—C14—C18	−58.6 (8)	H17B—C17—C19—O10	76.9 (11)
H16C—N16—C14—C18	179.5 (5)	N11B—C17—C19—O10	165.1 (12)
H16A—N16—C14—C18	63.0 (6)	N11—C17—C19—O10	−160.7 (4)
H11E—N11B—C17—H17A	43.9	H17A—C17—C19—O9	138.5 (8)
H11F—N11B—C17—H17A	−76.1	H17B—C17—C19—O9	−102.5 (10)
H11D—N11B—C17—H17A	163.9	N11B—C17—C19—O9	−14.3 (12)
H11E—N11B—C17—H17B	−64.0	N11—C17—C19—O9	19.9 (5)
H11F—N11B—C17—H17B	176.0	H21C—N21—C20—H20B	−175.1 (11)
H11D—N11B—C17—H17B	56.0	H21B—N21—C20—H20B	−54.8 (10)
H11E—N11B—C17—C19	−172.1	H21A—N21—C20—H20B	62.2 (11)
H11F—N11B—C17—C19	67.9	H21C—N21—C20—H20A	−57.3 (12)
H11D—N11B—C17—C19	−52.1	H21B—N21—C20—H20A	63.0 (11)
H11B—N11—C17—H17A	167.0 (11)	H21A—N21—C20—H20A	−180.0 (11)
H11A—N11—C17—H17A	−74.9 (11)	H21C—N21—C20—C13	64.1 (7)
H11C—N11—C17—H17A	46.3 (11)	H21B—N21—C20—C13	−175.6 (6)
H11B—N11—C17—H17B	49.3 (11)	H21A—N21—C20—C13	−58.6 (8)
H11A—N11—C17—H17B	167.4 (11)	O4—C13—C20—H20B	−120.9 (10)
H11C—N11—C17—H17B	−71.4 (11)	O3—C13—C20—H20B	59.2 (10)
H11B—N11—C17—C19	−72.8 (8)	O4—C13—C20—H20A	120.5 (10)
H11A—N11—C17—C19	45.3 (8)	O3—C13—C20—H20A	−59.4 (10)
H11C—N11—C17—C19	166.5 (7)	O4—C13—C20—N21	−0.9 (5)
H15—O15—C18—O7	−5.7 (10)	O3—C13—C20—N21	179.2 (3)

Symmetry codes: (i) −*x*+1, *y*+1/2, −*z*; (ii) −*x*+1, *y*−1/2, −*z*.

### Bis(glycinium) sulfate–glycine (1/1) (298KModel2) Hydrogen-bond geometry (Å, º)

**Table:**

*D*—H···*A*	*D*—H	H···*A*	*D*···*A*	*D*—H···*A*
O3—H3···O15^i^	1.06 (4)	1.41 (4)	2.451 (7)	166 (2)
O9—H9···O5^iii^	1.029 (7)	1.493 (7)	2.522 (5)	176.7 (8)
O15—H15···O3^ii^	1.07 (1)	1.39 (1)	2.451 (7)	178 (1)
N11—H11*A*···O3^iv^	1.022 (13)	1.800 (13)	2.809 (6)	168.3 (10)
N11—H11*B*···O10^iv^	1.011 (14)	1.943 (12)	2.807 (6)	141.7 (13)
N11—H11*C*···O2^v^	1.050 (11)	1.706 (11)	2.755 (5)	177.5 (8)
N11—H11*C*···O12^v^	1.050 (11)	2.527 (12)	3.132 (6)	116.0 (8)
N16—H16*A*···O6^vi^	1.036 (10)	1.815 (11)	2.785 (4)	154.2 (9)
N16—H16*B*···O2	1.019 (10)	2.074 (10)	2.907 (3)	137.5 (10)
N16—H16*B*···O4^vii^	1.019 (10)	2.333 (14)	2.919 (6)	115.4 (8)
N16—H16*C*···O5^iii^	1.026 (8)	2.258 (10)	2.974 (5)	125.5 (7)
N16—H16*C*···O12^iii^	1.026 (8)	1.982 (9)	2.974 (5)	161.6 (10)
N21—H21*A*···O6^vi^	1.019 (11)	1.880 (12)	2.829 (4)	153.6 (10)
N21—H21*B*···O2^iii^	1.017 (8)	2.076 (9)	3.024 (5)	154.3 (10)
N21—H21*B*···O5^iii^	1.017 (8)	2.213 (10)	2.969 (5)	129.9 (7)
N21—H21*C*···O12^viii^	1.015 (10)	1.989 (10)	2.892 (4)	146.9 (10)
N21—H21*C*···O7^vi^	1.015 (10)	2.516 (13)	3.007 (6)	109.3 (7)
C14—H14*A*···O10	1.082 (11)	2.402 (13)	3.287 (7)	138.0 (9)
C17—H17*A*···O7^ix^	1.062 (11)	2.583 (13)	3.404 (5)	133.8 (8)
C17—H17*B*···O4^x^	1.070 (11)	2.307 (12)	3.350 (5)	164.2 (12)
C20—H20*A*···O15^ix^	1.076 (15)	2.423 (15)	3.348 (7)	143.3 (11)
C20—H20*B*···O10	1.068 (13)	2.518 (14)	3.468 (7)	147.7 (10)

Symmetry codes: (i) −*x*+1, *y*+1/2, −*z*; (ii) −*x*+1, *y*−1/2, −*z*; (iii) −*x*+2, *y*+1/2, −*z*+2; (iv) *x*, *y*, *z*+1; (v) −*x*+1, *y*+1/2, −*z*+2; (vi) −*x*+2, *y*+1/2, −*z*+1; (vii) −*x*+2, *y*−1/2, −*z*+1; (viii) *x*, *y*+1, *z*; (ix) −*x*+1, *y*+1/2, −*z*+1; (x) −*x*+1, *y*−1/2, −*z*+1.
